# Particle Engineering via Supramolecular Assembly of Macroscopic Hydrophobic Building Blocks

**DOI:** 10.1002/anie.202315297

**Published:** 2023-12-20

**Authors:** Chan‐Jin Kim, Eirini Goudeli, Francesca Ercole, Yi Ju, Yuang Gu, Wanjun Xu, John F. Quinn, Frank Caruso

**Affiliations:** ^1^ Department of Chemical Engineering The University of Melbourne Parkville Victoria 3010 Australia; ^2^ Drug Delivery Disposition and Dynamics Theme Monash Institute of Pharmaceutical Sciences Monash University Parkville Victoria 3052 Australia; ^3^ School of Science RMIT University Melbourne Victoria 3000 Australia; ^4^ Department of Chemical Engineering Faculty of Engineering Monash University Clayton Victoria 3800 Australia

**Keywords:** Cell Association, Hydrophobic Effect, Particle Engineering, Physicochemical Property, Polymers

## Abstract

Tailoring the hydrophobicity of supramolecular assembly building blocks enables the fabrication of well‐defined functional materials. However, the selection of building blocks used in the assembly of metal–phenolic networks (MPNs), an emerging supramolecular assembly platform for particle engineering, has been essentially limited to hydrophilic molecules. Herein, we synthesized and applied biscatechol‐functionalized hydrophobic polymers (poly(methyl acrylate) (PMA) and poly(butyl acrylate) (PBA)) as building blocks to engineer MPN particle systems (particles and capsules). Our method allowed control over the shell thickness (e.g., between 10 and 21 nm), stiffness (e.g., from 10 to 126 mN m^−1^), and permeability (e.g., 28–72 % capsules were permeable to 500 kDa fluorescein isothiocyanate‐dextran) of the MPN capsules by selection of the hydrophobic polymer building blocks (PMA or PBA) and by controlling the polymer concentration in the MPN assembly solution (0.25–2.0 mM) without additional/engineered assembly processes. Molecular dynamics simulations provided insights into the structural states of the hydrophobic building blocks during assembly and mechanism of film formation. Furthermore, the hydrophobic MPNs facilitated the preparation of fluorescent‐labeled and bioactive capsules through postfunctionalization and also particle–cell association engineering by controlling the hydrophobicity of the building blocks. Engineering MPN particle systems via building block hydrophobicity is expected to expand their use.

## Graphical abstract

Introducing hydrophobic polymer building blocks enables the engineering of metal–phenolic network (MPN) particle properties (shell thickness, stiffness, permeability), and facilitates incorporation of functional molecules and control of cell association behavior. This study demonstrates the rational design and fabrication of hydrophobic MPNs with expanded utility for diverse applications.

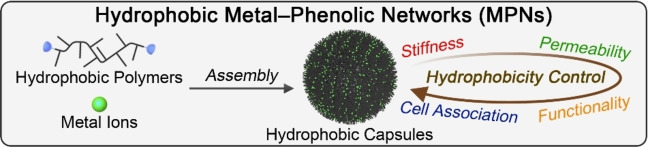

## Introduction

Supramolecular assembly enables the synthesis of functional materials with well‐defined structures and physicochemical properties by using a wide range of molecular building blocks such as block copolymers,^[1]^ peptide amphiphiles,^[2]^ oligonucleotides,^[3]^ and polyphenols.^[4]^ Furthermore, engineering hydrophobicity into these building blocks has enabled the fabrication of diverse assemblies such as self‐assembled polymeric micelles,[^1a^, ^1b^, ^1c^, ^1d^, ^1e^, ^1f^] peptide assemblies,^[2]^ DNA nanostructures,^[3]^ core–shell particles,[^4^, ^5^] and hollow capsules.^[4]^ Moreover, complexed/ordered structures displaying higher structural stability,[^1b^, ^3b^, ^4c^] controllable packing densities,[^1a^, ^1e^] morphology transitions,[^1f^, ^2c^] and efficient delivery[^1d^, ^1g^, ^2b^] can be obtained through the introduction of additional noncovalent (i.e., hydrophobic) interactions, which is useful for application in fields ranging from materials science to environmental and biomedical engineering.

The application of metal–phenolic networks (MPNs), which are robust and modular metal–organic assemblies governed by the coordination chemistry between metal ions and phenolic molecules (e.g., catechol and galloyl groups), has emerged as a versatile strategy for engineering particle systems through the deposition of MPNs on various substrates.^[6]^ The properties of MPN particle systems such as permeability,^[7]^ endosomal escape,^[8]^ and temperature‐induced structural rearrangement^[9]^ have been reported to be directly influenced by the selection of different phenolic molecules (e.g., tannic acid, gallic acid, pyrogallol, and quercetin). However, most MPN building blocks are currently limited to naturally occurring ligands. Thus, broadening the range of functional phenolic building blocks beyond commercially available phenolic compounds is expected to expand the range of physicochemical properties exhibited by MPN particle systems. Inspired by this, we recently reported the preparation of MPN capsules with tailorable properties (e.g., thickness, permeability, and cell association) using catechol‐functionalized poly(ethylene glycol) (PEG) blocks with different architectures and molecular weights.^[10]^ Furthermore, functional MPN particle systems displaying thermoresponsiveness or programmability (in terms of the hybridization between complementary DNA sequences) were fabricated using biscatechol‐functionalized thermoresponsive polymer building blocks (i.e., poly(*N*‐isopropylacrylamide),^[11]^ multi catechol‐functionalized copolymers (i.e., brush‐PEG),^[12]^ and catechol‐functionalized DNA block copolymers.^[13]^ Although well‐defined functional MPN particle systems have been fabricated using diverse building blocks (i.e., from naturally occurring molecules to synthetic polymers), these building blocks have essentially been hydrophilic in nature. Expanding the suite of MPN building blocks to utilize hydrophobic compounds will improve the versatility of MPN particle systems by providing additional and controllable noncovalent (i.e., hydrophobic) interactions. The hydrophobic properties of particle surfaces are also expected to be regulated via the choice of the hydrophobic building block employed and the development of MPN particles using a combination of hydrophobic and hydrophilic building blocks of varying ratios.

Herein, we demonstrate the fabrication of MPN capsules and particles with controlled properties using hydrophobic building blocks, guided by molecular dynamics (MD) simulations. Biscatechol‐functionalized hydrophobic polymers (based on poly(methyl acrylate) (PMA) and poly(butyl acrylate) (PBA)) having different hydrophobicities^[14]^ were designed and synthesized. The synthetic hydrophobic polymers were then employed to fabricate hydrophobic MPN capsules that were stable in various aqueous solutions and organic solvents owing to the coexisting interactions between the building blocks (i.e., metal coordination and hydrophobic interactions). The shell thickness, stiffness, and permeability of the MPN capsules were dependent on the hydrophobicity of the building blocks and the concentration of the hydrophobic building blocks in solution. The hydrophobic MPN capsules were functionalized with different dyes (e.g., fluorescein isothiocyanate‐tagged dextran (FITC‐dextran, green), rhodamine 6G (yellow), and rhodamine B (red)) or with functional proteins (i.e., horseradish peroxidase (HRP)) through hydrophobic interactions with the surface of the capsules. MPN particles prepared using different ratios of hydrophobic and hydrophilic polymers (i.e., biscatechol‐PMA and biscatechol‐poly(PEG methyl ether acrylate) (P(PEGA)) in the assembly solution afforded control of the cell association properties of the particles. This study demonstrates the rational design and fabrication of hydrophobic MPN capsules, (bio)functional MPN capsules, and MPN particles (prepared using a mixture of hydrophilic and hydrophobic polymer building blocks), which facilitates the preparation of tailor‐made particle systems with specific properties, and expands the use of hydrophobic polymers in MPNs for engineering materials and surfaces.

## Results and Discussion

**Figure 1:**
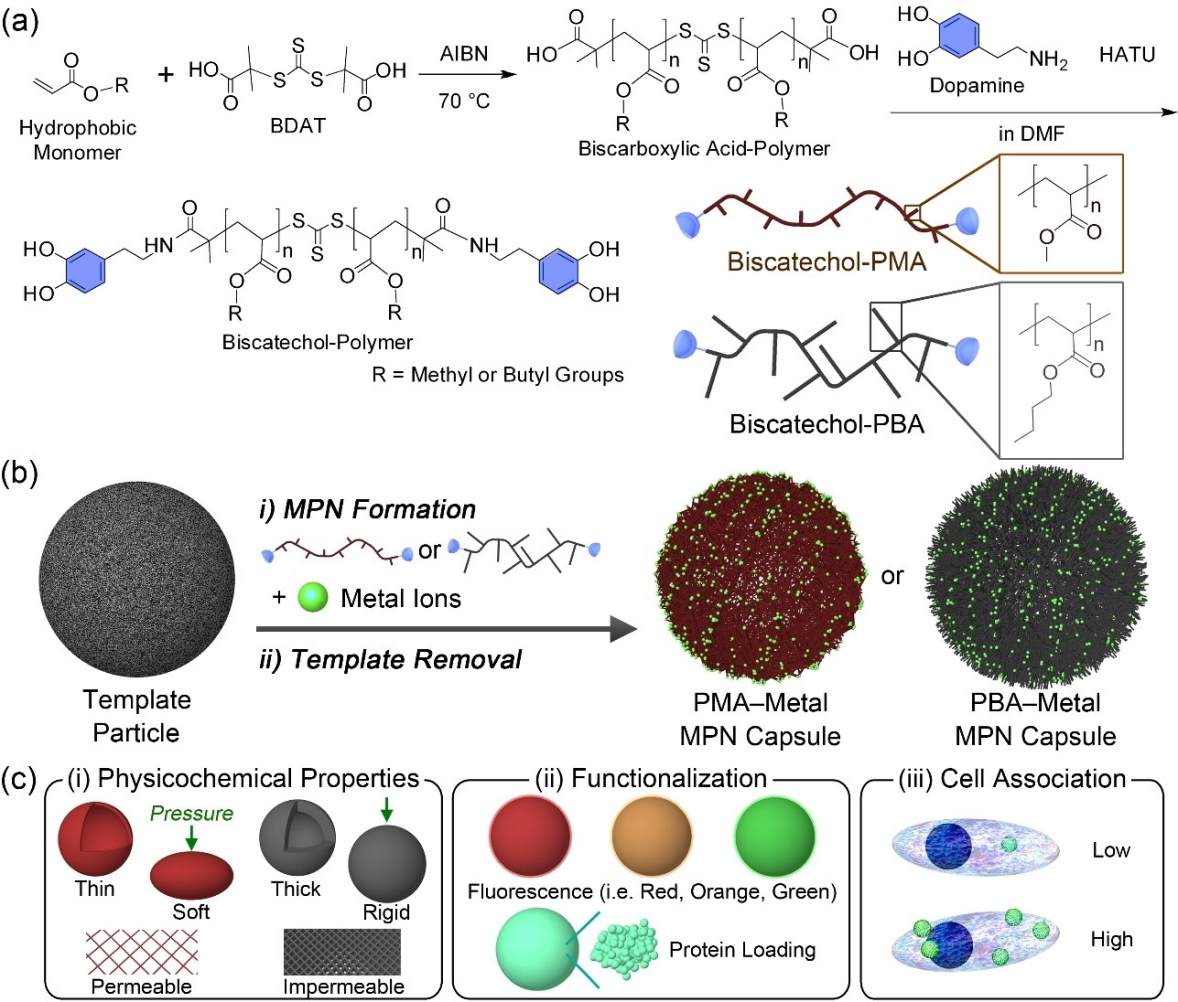
(a) Synthesis of biscatechol‐functionalized hydrophobic polymers. (b) Schematic illustration of the preparation of hydrophobic polymer‐metal MPN capsules using metal ions and biscatechol‐functionalized PMA or PBA and (c) subsequent engineering of their (i) physicochemical properties, (ii) functionalization/protein loading, and (iii) cell association properties. AIBN, 2,2′‐azobis(2‐methylpropionitrile); HATU, hexafluorophosphate azabenzotriazole tetramethyl uronium.

To assemble MPN particle systems with variable hydrophobicity, we first designed and synthesized a range of biscatechol‐functionalized polymers (biscatechol‐polymers) via reversible addition–fragmentation chain transfer (RAFT) polymerization, followed by amide bond formation (Figure 1a). To this end, a biscarboxyl acid‐terminated trithiocarbonate chain transfer agent (CTA), bis(α,α′‐dimethyl‐α′′‐acetic acid)‐trithiocarbonate (BDAT), was synthesized and characterized by nuclear magnetic resonance (1H NMR) spectroscopy (Figure S1).^[15]^ Thereafter, α,ω‐biscarboxylic acid‐functionalized polymers (biscarboxylic acid‐polymers) were synthesized by RAFT polymerization using BDAT as the CTA and 2,2′‐azobis(2‐methylpropionitrile) as the initiator. We selected three hydrophobic monomers—i.e., methyl acrylate (MA), butyl acrylate (BA), and styrene (St) with hydrophobicity varying as St>BA>MA)^[14]^—to afford MPNs with variable properties. A hydrophilic monomer—i.e., PEGA—was also selected to enable further elucidation of the influence of building block hydrophobicity on MPN properties. Hence, four different polymers were synthesized: biscarboxylic acid‐PMA_100_ (number‐average molecular weight (*M*_n_)=8900 g mol^−1^, repeating unit (*n*)=100); biscarboxylic acid‐PBA_118_ (*M*_n_=15 400 g mol^−1^, *n*=118); biscarboxylic acid‐PS_94_ (*M*_n_=10 100 g mol^−1^, *n*=94; PS denotes polystyrene); and biscarboxylic acid‐P(PEGA)_58_ (*M*_n_=28 300 g mol^−1^, *n*=58). The synthesized polymers were characterized by 1H NMR spectroscopy and gel permeation chromatography (Figures S2 and S3), and their properties are summarized in Table S1. The biscarboxylic acid‐polymers were then converted to biscatechol‐polymers via amide coupling with dopamine (at a high polymer/dopamine ratio (i.e., 1 : 10)) using hexafluorophosphate azabenzotriazole tetramethyl uronium, as characterized by 1H NMR spectroscopy (Figure S4). The peaks from coupled dopamine (i.e., 6.4–6.6 ppm) facilitated estimation of the coupling efficiencies between the biscatechol‐polymers and dopamine molecules, which are slightly higher than 100 %, indicating essentially complete conjugation (Table S2). As the peaks from conjugated dopamine overlapped with the broad aromatic peaks of PS (i.e., 6.4–7.2 ppm) (Figure S4c), biscatechol‐PS_94_ was further characterized by Fourier transform infrared spectroscopy (Figure S5). The appearance of the characteristic phenol O−H stretching band at 3534 cm^−1^ in the spectrum of biscatechol‐PS_94_, which was absent in the spectrum of biscarboxylic acid‐PS_94_, confirmed the successful synthesis of biscatechol‐PS_94_.^[16]^

Hydrophobic MPN capsules were fabricated via the formation of coordination networks between the synthesized biscatechol‐hydrophobic polymers (i.e., PMA, PBA, and PS) and metal ions (e.g., Fe^III^, Al^III^, Mn^II^, Cr^III^, and Cu^II^) on sacrificial templates (i.e., 1.86 μm PS‐COOH particles) (Figure 1b). Briefly, PS‐COOH particles, dispersed in a water/dimethyl formamide (DMF) mixture (DMF content=24 %), were mixed with biscatechol‐polymer solution and FeCl_3_ ⋅ 6H_2_O. 3‐(*N*‐Morpholino)propanesulfonic acid (MOPS) buffer (25 mM, pH 7.4) was then added to the suspension to raise the pH (DMF content=10 %), resulting in bis‐ and tris‐dominant coordination states between biscatechol‐polymers and Fe^III^ ions.^[6a]^ Selective dissolution of the PS−COOH particles with tetrahydrofuran yielded hydrophobic MPN capsules.^[11]^ In the present study, we mainly focused on PMA−Fe^III^ and PBA−Fe^III^ MPN capsules to compare the effect of varying the hydrophobic polymer building block on capsule properties as PS−Fe^III^ MPN capsules were prone to aggregation. The physicochemical properties of the synthesized MPN capsules, such as shell thickness, stiffness, and permeability, were tuned by the polymer type and concentration of the polymer assembly solution. Furthermore, fluorescence labeling and protein loading of the capsules were achieved by postfunctionalization, exploiting the noncovalent bonding interactions (i.e., hydrophobic interactions) between the functional molecules and the capsule surface. Regulating the ratio of hydrophobic and hydrophilic building blocks enabled tuning of the cell association properties of MPN particles (Figure 1c).

**Figure 2:**
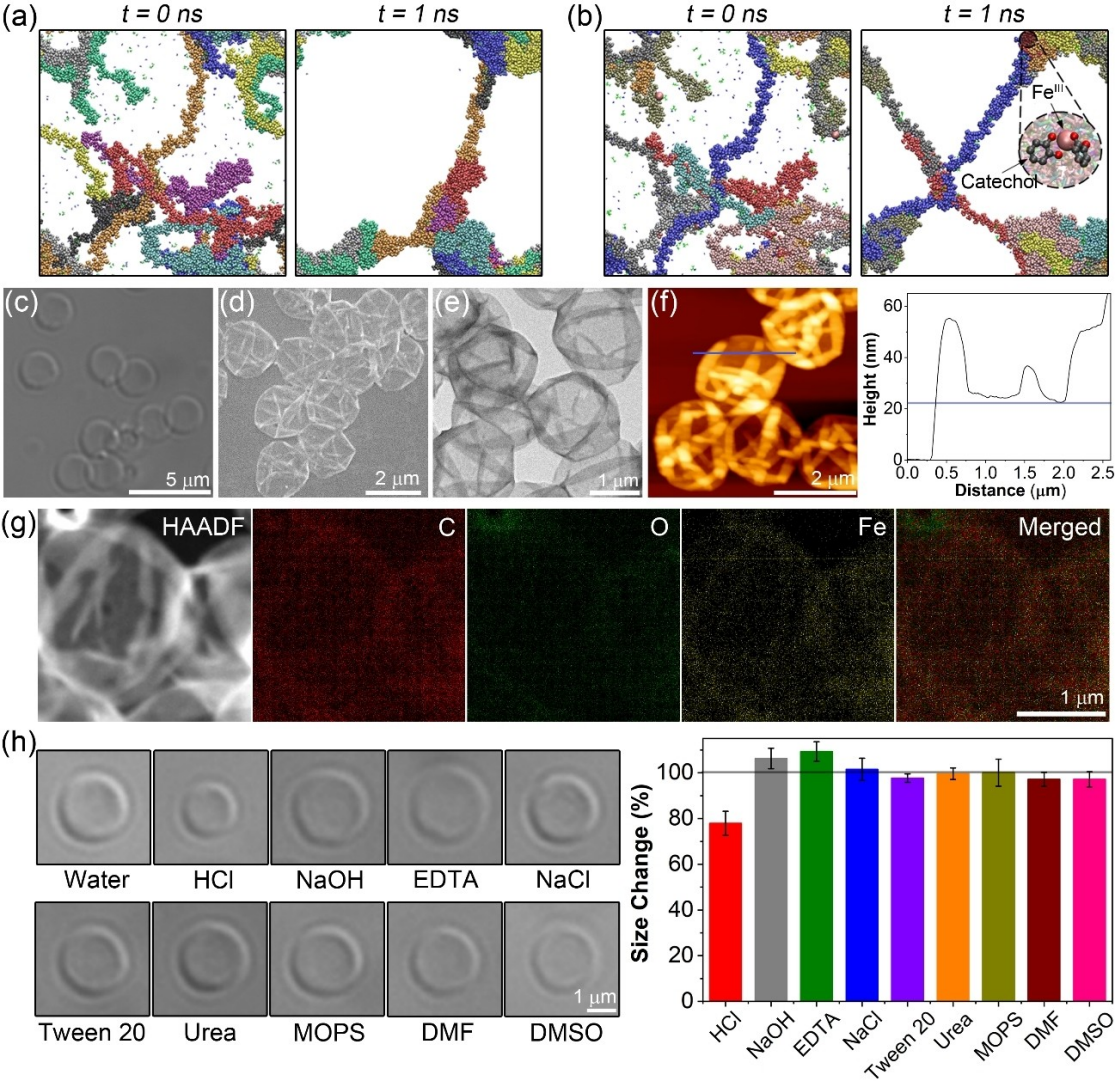
MD simulation snapshots of (a) PMA chains and (b) PMA−Fe^III^ complexes in water/DMF mixture (DMF content=24 %) at *t*=0 and 1 ns. Polymer chains are color‐coded. Characterization of PMA_100_−Fe^III^ MPN capsules, prepared using 0.5 mM polymer solution at a constant catechol/Fe^III^ ion ratio of 1 : 1, via (c) DIC, (d) SEM, (e) TEM, (f) AFM, (g) HAADF, and EDX elemental mapping. The height‐distance profile of an MPN capsule plotted along the blue line in the AFM image is also shown. (h) Stability of PMA_100_−Fe^III^ MPN capsules in different environments. Representative DIC images of capsules after incubation for 1 h in water (control), 0.5 M HCl, 0.5 M NaOH, 100 mM EDTA, 100 mM NaCl, 100 mM Tween 20, 100 mM urea, 50 mM MOPS (pH 8), DMF, and DMSO. Variations in size of PMA_100_−Fe^III^ MPN capsules following incubation for 1 h in different solutions, as determined from DIC measurements of 20 capsules. Changes in size are calculated relative to the capsule size measured in water (100 %).

Hydrophobic polymer building blocks exist in the coil or globular state depending on the content ratio between water and good solvent (e.g., DMF). To understand the states of the biscatechol‐polymers during MPN assembly, MD simulations were performed (Figures 2a and S6a). The biscatechol‐polymers adopted a coil structure in dilute water/DMF mixtures (DMF content=24 %), which facilitated the formation of MPNs. Furthermore, the simulation studies indicated that the MPN films assembled through hydrophobic interactions as well as metal coordination networks, with hydrophobic interactions appearing to be the more dominant force when hydrophobic building blocks are used for assembly (Figures 2b and S6b). For example, the decrease in the peak of the radial distribution function between biscatechol‐PMA and water in the presence of Fe^III^ ions suggests a more dominant role of hydrophobic interactions (Figure S7).

PMA_100_−Fe^III^, PBA_118_−Fe^III^, and P(PEGA)_58_−Fe^III^ MPN capsules, prepared using polymer assembly solutions with concentrations ranging from 0.25 to 2.0 mM and a catechol/Fe^III^ ion ratio of 1 : 1, were characterized by differential interference contrast (DIC) microscopy, scanning electron microscopy (SEM), energy‐dispersive X‐ray spectroscopy (EDX), transmission electron microscopy (TEM), atomic force microscopy (AFM), and high‐angle annular dark‐field (HAADF) microscopy (Figures 2c–g, S8–S10). These images showed well‐dispersed capsules in solution and typical capsule structures in the air‐dried state (i.e., with evident folds and creases). The shell thickness of PMA_100_−Fe^III^ MPN capsules prepared using a 0.5 mM polymer assembly solution (denoted as 0.5 mM PMA_100_−Fe^III^ MPN capsules) was 12.0±0.7 nm, as determined via the minimum capsule thickness from AFM measurements. EDX elemental mapping revealed that C, O, and Fe were uniformly distributed in the capsules, indicating that the PMA_100_−Fe^III^ MPN capsules were composed of PMA_100_ building blocks and Fe^III^ ions. PS_94_−Fe^III^ MPN capsules, prepared using the polymer with the highest hydrophobicity among those examined in the present study,^[14]^ showed evidence of aggregation when the concentration of the polymer assembly solution was higher than 0.5 mM, likely due to the strong hydrophobic interactions between the PS building blocks (Figure S8). This result emphasizes that the proper selection of hydrophobic MPN building block and assembly conditions are important to obtain well‐dispersed capsules. The versality of the synthesis method is demonstrated through the preparation of MPN capsules containing different metal ions (e.g., Al^III^, Mn^II^, Cr^III^, and Cu^II^) (Figure S11). Furthermore, by using two different polymer building blocks such as PMA/P(PEGA) (1 : 1), PBA/P(PEGA) (1 : 1), or PS/P(PEGA) (1 : 1) (i.e., a hydrophobic polymer and a hydrophilic polymer), we demonstrated the feasibility of preparing hybrid polymer–Fe^III^ MPN capsules (Figure S12).

Subsequently, we investigated the stability of the PMA_100_−Fe^III^ MPN capsules in various solutions: acidic (0.5 M HCl), alkaline (0.5 M NaOH), metal‐chelating (100 mM ethylenediaminetetraacetic acid (EDTA)), high ionic strength (e.g., 100 mM NaCl), surfactant (100 mM Tween 20), hydrogen bonding competitor (100 mM urea), buffered (50 mM MOPS (pH 8)), and organic (e.g., DMF and dimethyl sulfoxide (DMSO)) (Figures 2h and S13). The results indicated that the PMA_100_−Fe^III^ MPN capsules displayed a high structural stability, likely due to the dual assembly interactions in the capsules (i.e., hydrophobic interactions and metal coordination bonds). The size of the capsules was influenced by the surrounding environment, likely due to the protonation and deprotonation of the catechol groups in the polymer building blocks and the extent of metal coordination interactions. For instance, relative to the capsule size measured in water, the capsule size in HCl decreased by 22 %, whereas that in NaOH and EDTA increased by 6 % and 9 %, respectively.

**Figure 3:**
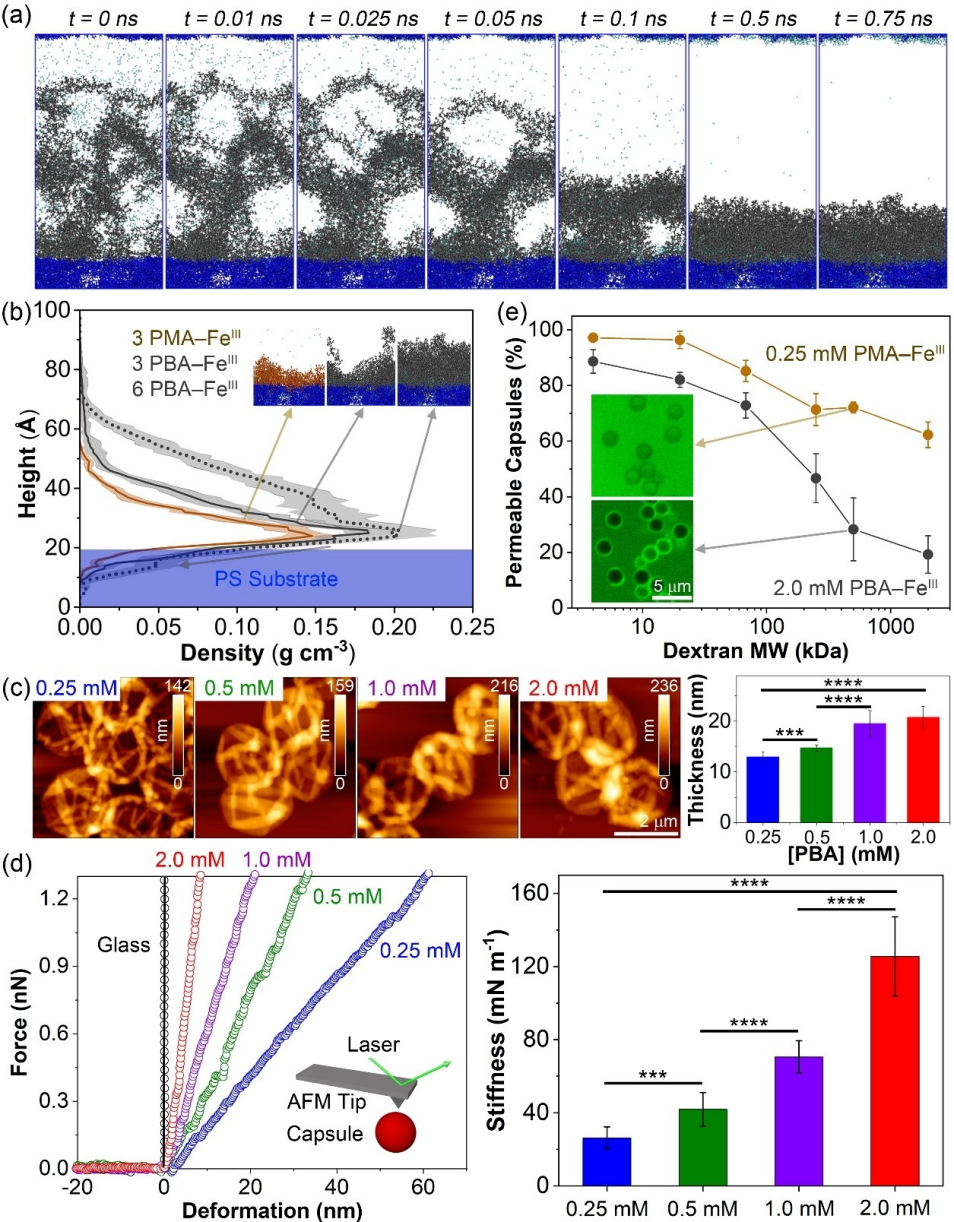
(a) MD simulation snapshots of a biscatechol‐PBA−Fe^III^ film (6 strands of PBA and 3 Fe^III^ ions) depositing on a PS substrate. (b) Density distribution of polymer−Fe^III^ films (3 or 6 strands of PMA or PBA with 3 Fe^III^ ions) as a function of height from the PS substrate. (c) Shell thickness of PBA_118_−Fe^III^ MPN capsules prepared using different PBA concentrations in the assembly solution. The shell thickness was determined by height‐distance AFM analysis and is shown as the mean ± standard deviation of 10 independent AFM measurements. (d) Representative force‐deformation (*F*–*δ*) curves and corresponding stiffness profiles of the PBA_118_−Fe^III^ MPN capsules prepared using assembly solutions with PBA concentrations ranging from 0.25 to 2.0 mM. The *F*–*δ* curve of a glass substrate is also shown for comparison. The data are shown as mean stiffness±standard deviation (*n*=10). Statistical significance was determined by one‐way analysis of variance (ANOVA): ****p<0.0001 and ***p<0.001. (e) Permeability of PMA_100_−Fe^III^ or PBA_118_−Fe^III^ MPN capsules to FITC‐dextran with *M*_w_ ranging from 4 to 2000 kDa and corresponding representative confocal microscopy images (inset, scale bars are 5 μm). The permeability data are shown as the mean ± standard deviation of three independent experiments; 50–100 capsules were examined.

The mechanism of MPN film formation based on hydrophobic polymer−Fe^III^ complexes was investigated by MD simulations (Figures 3a, 3b, and S14). To simplify the simulation calculation, a substrate composed of 10 PS chains was constructed with different concentrations (3 and 6 chains) of biscatechol‐PMA−Fe^III^ and biscatechol‐PBA−Fe^III^ complexes randomly distributed within 15 nm of the PS substrate. The biscatechol‐PBA−Fe^III^ chains fill the free space above the PS substrate within a relatively longer timeframe (*t*=0.5 ns) than the biscatechol‐PMA−Fe^III^ chains, leading to thicker and denser films. Moreover, higher concentrations of polymer−Fe^III^ complexes resulted in thicker films. For instance, 6 PBA−Fe^III^ complexes resulted in ≈30 % thicker films when compared with 3 PBA−Fe^III^, as illustrated by the broader density distribution of the 6 PBA−Fe^III^ complexes (Figure 3b).

The discrete MPN assembly method generally provides capsules with a shell thickness of around 10 nm, regardless of the type of metal ion or phenolic molecule employed.[^6a^, ^17^] Engineering the shell thickness can provide a level of control over the physicochemical properties of the capsules, such as stiffness and permeability, which can be useful for materials science and biomedical applications.[^17^, ^18^] However, controlling the MPN film thickness is difficult owing to kinetic trapping and symmetry breaking at the interface over a short time (<1 min), resulting in the termination of film growth.^[19]^ Employing oxidation‐mediated processes,^[19]^ continuous assembly,^[17]^ and repeated MPN assembly cycles,^[18a]^ and customizing the hydrophilic phenolic ligand blocks with different structures and molecular weights^[10]^ are alternative methods for engineering the shell thickness of MPN capsules. Although these methods can be used to control the film thickness, they typically require long assembly times (≈4 h), multiple coating steps, and potentially laborious synthesis procedures to prepare different building blocks. Alternatively, in the present study, guided by MD simulation data, we proposed an alternative strategy to control shell thickness by regulating the concentration (0.25–2.0 mM) of the hydrophobic MPN building block (i.e., biscatechol‐PBA) assembly solution, while maintaining a constant catechol/Fe^III^ ion ratio of 1 : 1. A quantitative comparison of the shell thickness of the capsules by AFM revealed that the shell thickness of the PBA_118_−Fe^III^ MPN capsules increased from 13 to 21 nm as the polymer concentration in the assembly solution increased from 0.25 to 2.0 mM (Figure 3c). Higher PBA concentrations in solution likely result in larger aggregates being formed owing to hydrophobic interactions, with thicker MPN films being deposited upon metal coordination. PMA_100_−Fe^III^ MPN capsules followed a similar shell thickness trend to the PBA_118_−Fe^III^ MPN capsules (Figure S10b), although the higher hydrophobicity of PBA compared with that of PMA generally resulted in thicker films (e.g., 20.8±2.0 nm for 2.0 mM PBA_118_−Fe^III^ MPN capsules vs 15.5±1.1 nm for 2.0 mM PMA_100_−Fe^III^ MPN capsules). These results indicate the significant role of hydrophobic interactions between building blocks in regulating the shell thickness of the MPN capsules. In contrast, the shell thickness of the P(PEGA)_58_−Fe^III^ MPN capsules (prepared with the hydrophilic polymer block P(PEGA)) was unaffected by the concentration of polymer in the assembly solution, likely due to the absence of hydrophobic interactions in this MPN system in the assembly process. The mechanical properties of the PBA_118_−Fe^III^ MPN capsules were investigated by obtaining force‐deformation (*F*–*δ*) curves via AFM force measurement analysis (Figure 3d). The stiffness values, determined from the slope of the curves,[^11^, ^17^, ^18^, ^20^] were 26±6, 42±9, 71±9, and 126±22 mN m^−1^ for the 0.25, 0.5, 1.0, and 2.0 mM PBA_118_−Fe^III^ MPN capsules, respectively. The increase in stiffness from soft to rigid capsules observed with increasing MPN building block concentrations was likely due to the formation of thicker and denser film states. The stiffness of the PMA_100_−Fe^III^ MPN capsules also showed a similar trend, increasing from 10 to 63 mN m^−1^ as the PMA concentration increased (Figure S15). The relatively lower stiffness values of the PMA_100_−Fe^III^ MPN capsules compared with those of the PBA_118_−Fe^III^ MPN capsules were likely due to the differences in shell thickness of these capsules.

We subsequently investigated the permeability of the PMA_100_−Fe^III^ and PBA_118_−Fe^III^ MPN capsules prepared from 0.25–2.0 mM polymer assembly solutions by incubating the capsules with FITC‐dextran of different molecular weights (*M*_w_s) ranging from 4 to 2000 kDa (Figures 3e, S16, and S17). As the radius of gyration increases with the *M*_w_ of FITC‐dextran, this also results in a decrease in permeability through the capsule wall. A decrease in permeability of capsules is reflective of a more dense network structure.[^10^, ^21^] Generally, PBA_118_−Fe^III^ MPN capsules were slightly less permeable than PMA_100_−Fe^III^ MPN capsules, which can be attributed to the denser network formed from PBA building blocks owing to their higher degree of hydrophobicity than PMA. The concentration of the MPN assembly solution also slightly influenced the permeability of the capsules. For example, the MPN capsules prepared from an assembly solution with 0.25 mM PMA were more permeable than those prepared from higher concentrations (i.e., 0.5–2.0 mM) (Figure S16). To provide a comparison of permeability, 0.25 mM PMA_100_−Fe^III^ MPN capsules and 2.0 mM PBA_118_−Fe^III^ MPN capsules were selected. As shown in Figure 3e, the permeability of 0.25 mM PMA_100_−Fe^III^ MPN capsules was higher than that of 2.0 mM PBA_118_−Fe^III^ MPN capsules. Specifically, 72 % of the 0.25 mM PMA_100_−Fe^III^ MPN capsules and 28 % of the 2.0 mM PBA_118_−Fe^III^ MPN capsules were permeable to 500 kDa FITC‐dextran, respectively. MD simulations were performed to study the pore size of these two different systems. For *t* >0.2 ns, the biscatechol‐PBA−Fe^III^ forms a structurally stable, less porous film (Figure S18) of constant thickness (Figure 3a) than the biscatechol‐PMA−Fe^III^ film.

**Figure 4:**
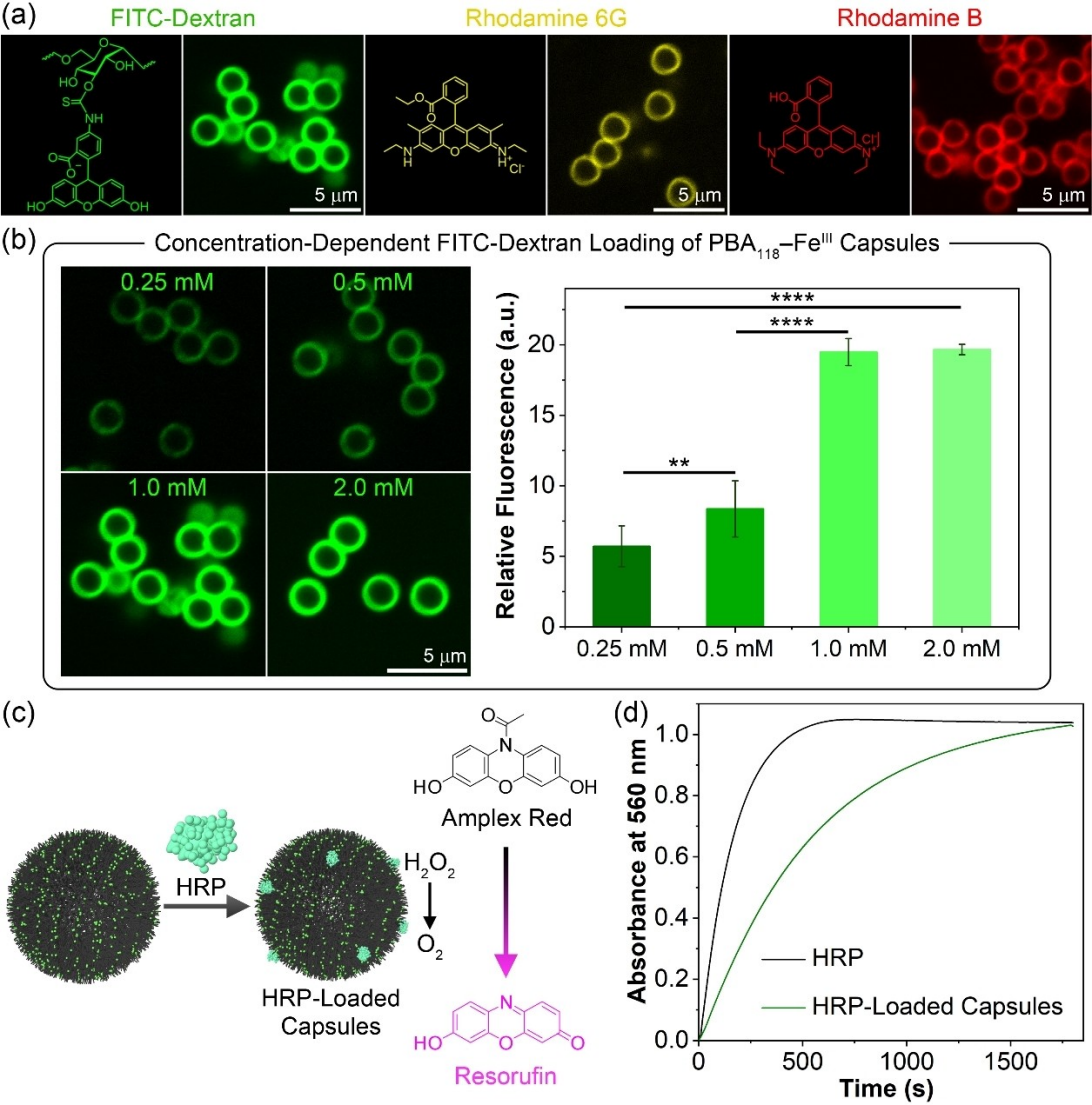
(a) CLSM images of PBA_118_−Fe^III^ MPN capsules loaded with fluorescent molecules i.e., FITC‐dextran (green), rhodamine 6G (yellow), or rhodamine B (red) after incubation for 5 min. (b) Effect of PBA concentration in the assembly solution on the loading of PBA_118_−Fe^III^ MPN capsules; incubation was performed for 5 min. The statistical significance was determined by one‐way ANOVA: ****p<0.0001 and **p<0.01. (c) Schematic illustration of the preparation of HRP‐loaded PBA_118_−Fe^III^ MPN capsules by postfunctionalization and their enzymatic activity. (d) Time‐dependent absorbance changes upon oxidation of amplex red, catalyzed by free HRP or HRP‐loaded PBA_118_−Fe^III^ MPN capsules.

Incorporating fluorescent molecules and bioactive macromolecules (i.e., proteins) into particle platforms is useful for bioimaging,^[22]^ catalysis,^[23]^ and therapeutic agent delivery.^[2b]^ Accordingly, herein, we examined the postfunctionalization of the MPN capsules in further extending their application. We hypothesized that the hydrophobic properties of the hydrophobic MPN capsules could enhance association with fluorescent dyes and functional proteins through hydrophobic effects. To investigate this, three fluorescent molecules (i.e., 2000 kDa FITC‐dextran (green), rhodamine 6G (yellow), rhodamine B (red)) were selected as model molecules and incubated separately with PBA_118_−Fe^III^ MPN capsules for 5 min. The resulting capsules exhibited bright green, yellow, and red fluorescence at their surfaces, as observed by confocal laser scanning microscopy (CLSM), which was indicative of physical association of the dyes via hydrophobic interactions (Figure 4a). In contrast, P(PEGA)_58_−Fe^III^ or PMA_100_−Fe^III^ MPN capsules, composed of hydrophilic or less hydrophobic MPN building blocks, showed weaker fluorescence owing to weaker interactions with the dye molecules (Figure S19). Cationic dyes such as rhodamine 6G and rhodamine B bound to these capsules due to the negatively charged surface of the MPN capsules (the ζ‐potential values are approximately −16 mV, approximately −25 mV, and approximately −22 mV for P(PEGA)_58_−Fe^III^, PMA_100_−Fe^III^, and PBA_118_−Fe^III^ MPN capsules, respectively). Subsequently, we investigated the loading of FITC‐dextran into PBA_118_−Fe^III^ MPN capsules prepared using assembly solutions of different concentrations of PBA building block (0.25–2.0 mM). PBA−Fe^III^ MPN capsules prepared using higher PBA building block concentrations (i.e., 1.0 and 2.0 mM) showed more pronounced fluorescence than those prepared using the lower PBA concentrations (Figure 4b). The stronger fluorescence observed was attributed to the presence of more PBA strands on the capsules binding to the fluorescent molecules. Furthermore, as expected, longer incubation times also resulted in higher dye loading into the MPN capsules (Figure S20). We also note that within 1 min of incubation with FITC‐dextran, the PBA_118_−Fe^III^ MPN capsules reached 50 % of the fluorescence intensity observed after incubation for 60 min, indicating rapid binding interactions between the capsule surface and dye molecules.

The loading of the PBA_118_−Fe^III^ MPN capsules with a functional protein (i.e., HRP) was also examined along with the catalytic activity of the resulting MPN capsules. HRP is known to catalyze the oxidative conversion of amplex red into resorufin (which has a strong absorbance at 560 nm) by H_2_O_2_ (Figure 4c).^[24]^ As observed from Figure 4d, the HRP‐loaded PBA_118_−Fe^III^ MPN capsules showed comparable catalytic activity to free HRP, indicating successful enzyme loading on the capsule surface, without significant loss of enzyme activity. These results demonstrate that the MPN capsules fabricated using hydrophobic building blocks (i.e., PBA−Fe^III^ MPN capsules) can be readily functionalized by functional molecules (e.g., dyes and proteins) through hydrophobic effects, which could be useful for biomedical applications.

**Figure 5:**
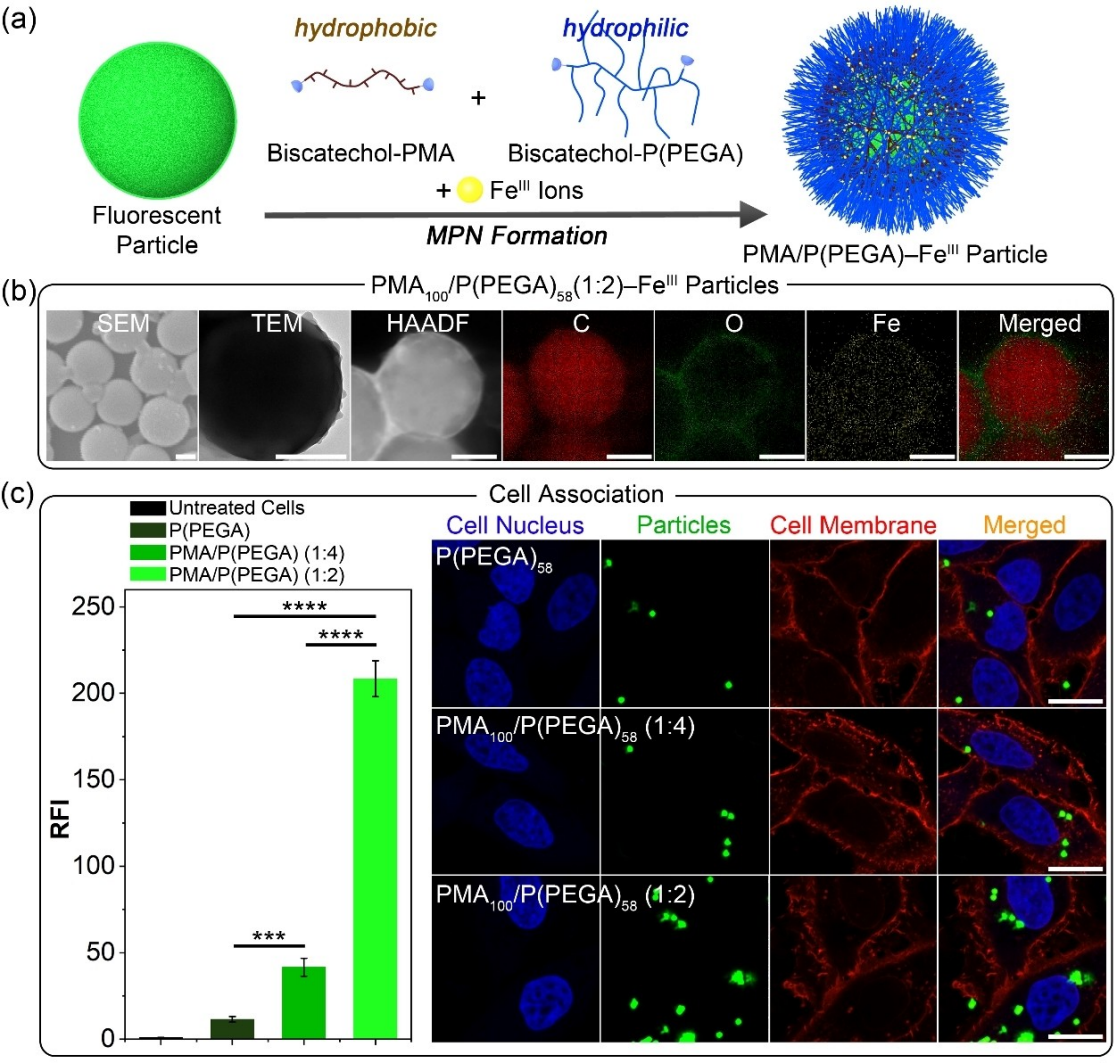
(a) Schematic illustration of the preparation of MPN particles using Fe^III^ ions, biscatechol‐PMA, and biscatechol‐P(PEGA). (b) SEM, TEM, HAADF, and EDX elemental mapping of PMA_100_/P(PEGA)_58_(1 : 2)−Fe^III^ MPN particles. Scale bars are 500 nm. (c) Cell association of P(PEGA)_58_−Fe^III^, PMA_100_/P(PEGA)_58_(1 : 4)−Fe^III^, and PMA_100_/P(PEGA)_58_(1 : 2)−Fe^III^ MPN particles, as assessed by flow cytometry (left) and confocal microscopy (right). HeLa cells were treated with MPN particles for 4 h at 37 °C at a cell‐to‐particle ratio of 1 : 10. The RFI to untreated cells is shown. The error bars represent the standard deviation of three independent experiments. The statistical significance was determined by one‐way ANOVA: ****p<0.0001 and ***p<0.001. CLSM images of HeLa cells incubated with MPN particles for 24 h at 37 °C. Cell membranes and nuclei were stained with WGA594 (red) and Hoechst 33342 (blue), respectively. The green fluorescence represents the MPN particles. Scale bars are 20 μm.

PEG has been widely exploited in biomedical applications owing to its stealth properties.^[25]^ PEG chains in biofunctional particle systems have been shown to reduce cytotoxicity and nonspecific interactions with cells and proteins, and provide well‐dispersed nonaggregated particles.^[26]^ In contrast, increasing the level of hydrophobicity in particle systems can enhance cell association as well as aggregation between particles.^[27]^ Therefore, maintaining an optimum hydrophilic‐hydrophobic balance for a particle system is important for efficient therapeutic delivery. We hypothesized that the synthesis of PMA/P(PEGA) MPN particles using biscatechol‐PMA_100_ (hydrophobic polymer) and biscatechol‐P(PEGA)_58_ (hydrophilic polymer) at varying PMA/P(PEGA) molar ratios in the assembly solution could facilitate control over the cell association of the particles. To compare the binding properties of the PMA/P(PEGA) MPN particles, highly fluorescent MPN particles were synthesized via an MPN coassembly method using a combination of hydrophobic and hydrophilic polymer strands at different ratios (Figure 5a). Briefly, green‐fluorescent PS‐COOH particles (1.20 μm) were coated by MPN films composed of different ratios of PMA_100_ and P(PEGA)_58_ (i.e., PMA/P(PEGA)=0 : 1, 1 : 4, and 1 : 2). The synthesized P(PEGA)_58_−Fe^III^, PMA_100_/P(PEGA)_58_(1 : 4)−Fe^III^, and PMA_100_/P(PEGA)_58_(1 : 2)−Fe^III^ MPN particles were characterized by DLS, SEM, TEM, HAADF, and EDX mapping (Figures 5b, S21–S23). DLS data indicated that all prepared particles were well dispersed (polydispersity index < 0.21) and the microscopy images revealed that both the P(PEGA) and PMA/P(PEGA) MPN films were homogeneously coated on the particle surfaces. EDX elemental mapping additionally showed that C, O, and Fe were uniformly distributed on the particles, indicating that the films are composed of polymer−Fe^III^ complexes. The ζ‐potential measurements showed that the prepared particles had similar negative ζ‐potentials (approximately −25±2 mV) for fluorescent particles prepared using PMA/P(PEGA) ratios of 0 : 1, 1 : 4, and 1 : 2.

The binding properties of the synthesized MPN particles (i.e., P(PEGA)_58_−Fe^III^, PMA_100_/P(PEGA)_58_(1 : 4)−Fe^III^, and PMA_100_/P(PEGA)_58_(1 : 2)−Fe^III^ MPN particles) were determined by analyzing their cell association properties by flow cytometry and CLSM (Figure 5c). The particles were incubated with HeLa cells for 4 h at 37 °C at a cell‐to‐particle ratio of 1 : 10. The relative fluorescence intensity (RFI) values to untreated cells for P(PEGA)_58_−Fe^III^, PMA_100_/P(PEGA)_58_(1 : 4)−Fe^III^, and PMA_100_/P(PEGA)_58_(1 : 2)−Fe^III^ MPN particles were measured as 11.5, 41.6, and 208.4, respectively, indicating that increasing the proportion of the hydrophobic component results in increased cell association. To visualize the cellular association of the particles, the cell nucleus and membrane were stained by Hoechst 33342 (blue) and wheat germ agglutinin (WGA)594 (red), which do not overlap with the fluorescence from the MPN particles (green). Consistent with the flow cytometry results, the PMA_100_/P(PEGA)_58_(1 : 2)−Fe^III^ MPN particles with the highest proportion of hydrophobic component among the three particles, showed the highest cell association. Additionally, all three particle systems showed negligible cytotoxicity, supporting their potential use in biomedical applications (Figure S24). These results demonstrate a simple approach for controlling the cell association of particles, simply by employing MPN assembly with hydrophilic and hydrophobic building blocks (i.e., P(PEGA) and PMA).

## Conclusion

We designed and synthesized biscatechol‐functionalized hydrophobic polymers (i.e., PMA and PBA) as building blocks for the fabrication of hydrophobic polymer−Fe^III^ MPN capsules. Simulation results demonstrated the structural states of the polymer blocks during assembly and revealed that the major interactions in these supramolecular MPN films included hydrophobic interactions and metal coordination. These coexisting interactions promoted the structural stability of the MPN capsules in various conditions, including low and high pH, high ionic strength, and the presence of a chelating agent, surfactant, buffer, or organic solvent. The properties of the capsules (i.e., shell thickness, stiffness, and permeability) could simply be tuned by varying the concentration of the hydrophobic polymer building block in the assembly solution and hydrophobicity of the selected polymer. This contrasts with conventional MPN complexes (prepared from natural polyphenol building blocks (which are largely hydrophilic)) that typically require additional engineering assembly steps or longer assembly times. The use of more hydrophobic polymer building blocks and higher concentrations of polymer in the assembly solution resulted in thicker, more rigid, and less permeable capsule shells. The synthesized hydrophobic capsules (i.e., PBA−Fe^III^ MPN capsules) were readily functionalized with different fluorescent and enzyme molecules; loading was facilitated through the formation of hydrophobic interactions, which is a key characteristic of the present MPN system over conventional MPN systems. Furthermore, the cell association of MPN particles (i.e., PMA/P(PEGA)−Fe^III^ MPN particles) could be controlled by varying the ratio of the hydrophobic (i.e., PMA) to the hydrophilic (i.e., P(PEGA)) building blocks in the assembly solution. Specifically, by increasing the relative amount of the hydrophobic polymer, the degree of cell association was increased. Collectively, the findings show that the incorporation of hydrophobic polymer building blocks into MPN particle systems enables control over the physicochemical properties of the particles, allowing for biofunctionality and regulation of the cell association behavior. We envision that the use of hydrophobic building blocks in MPNs could provide a strategy to precisely engineer particles for materials science and biomedical applications such as micro/nano reactors and delivery vehicles.

## Conflict of interest

The authors declare no conflict of interest.

1

## Supporting information

As a service to our authors and readers, this journal provides supporting information supplied by the authors. Such materials are peer reviewed and may be re‐organized for online delivery, but are not copy‐edited or typeset. Technical support issues arising from supporting information (other than missing files) should be addressed to the authors.

Supporting Information

## Data Availability

The data that support the findings of this study are available from the corresponding author upon reasonable request.
