# Supplementary material for: Particle Engineering via Supramolecular Assembly of Macroscopic Hydrophobic Building Blocks
Source: Angew Chem Int Ed Engl. 2023 Dec 20;63(4):e202315297. doi: 10.1002/anie.202315297 (PMC10953382; doi:10.1002/anie.202315297)
Supplement: Supplementary file 1 — Supporting Information [file ANIE-63-0-s001.pdf]

Angewandte Chemie
Eine Zeitschrift der Gesellschaft Deutscher Chemiker

Supporting Information

Particle Engineering via Supramolecular Assembly of Macroscopic Hydrophobic Building Blocks

*C.-J. Kim, E. Goudeli, F. Ercole, Y. Ju, Y. Gu, W. Xu, J. F. Quinn, F. Caruso**

Supporting Information
©Wiley-VCH 2021
69451 Weinheim, Germany

Particle Engineering via Supramolecular Assembly of Macroscopic Hydrophobic Building Blocks

Chan-Jin Kim, Eirini Goudeli, Francesca Ercole, Yi Ju, Yuang Gu, Wanjun Xu, John F. Quinn, and Frank Caruso*

Abstract: Tailoring the hydrophobicity of supramolecular assembly building blocks enables the fabrication of well-defined functional materials. However, the selection of building blocks used in the assembly of metal–phenolic networks (MPNs), an emerging supramolecular assembly platform for particle engineering, has been essentially limited to hydrophilic molecules. Herein, we synthesized and applied biscatechol-functionalized hydrophobic polymers (poly(methyl acrylate) (PMA) and poly(butyl acrylate) (PBA)) as building blocks to engineer MPN particle systems (particles and capsules). Our method allowed control over the shell thickness (e.g., between 10 and 21 nm), stiffness (e.g., from 10 to 126 mN m^{-1}), and permeability (e.g., 28–72% capsules were permeable to 500 kDa fluorescein isothiocyanate-dextran) of the MPN capsules by selecting hydrophobic polymer building blocks (PMA or PBA) and controlling the polymer concentration in the MPN assembly solution (0.25–2.0 mM) without additional/engineered assembly processes. Molecular dynamics simulations provided insights in the structural states of the hydrophobic building blocks during assembly and mechanism of film formation. Furthermore, the hydrophobic MPNs facilitated the preparation of fluorescent-labeled and bioactive capsules through postfunctionalization and particle–cell association engineering by controlling the hydrophobicity of the building blocks. Engineering MPN particle systems via building block hydrophobicity is expected to expand their use.

DOI: 10.1002/anie.2021XXXXX

Materials and Instrumentation

Carbon disulfide, tetrabutylammonium bisulfate, mineral spirits, sodium hydroxide, dopamine·HCl, hexafluorophosphate azabenzotriazole tetramethyl uronium (HATU), triethylamine (TEA), acetone, chloroform, *N*,*N*-dimethylformamide (DMF), dichloromethane, dimethyl sulfoxide (DMSO), butyl acrylate, methyl acrylate, poly(ethylene glycol) methyl ether acrylate (PEGA, M_n = 480 g mol^{-1}), styrene, 2,2′-azobis(2-methylpropionitrile) (AIBN), iron(III) chloride hexahydrate ($FeCl_3 \cdot 6H_2O$), aluminum(III) chloride ($AlCl_3$), manganese(II) sulfate monohydrate ($MnSO_4 \cdot H_2O$), chromium(III) chloride hexahydrate ($CrCl_3 \cdot 6H_2O$), copper(II) nitrate trihydrate ($Cu(NO_3)_2 \cdot 3H_2O$), 3-(*N*-morpholino)propanesulfonic acid (MOPS), tetrahydrofuran, ethylenediaminetetraacetic acid (EDTA), sodium chloride, Tween 20, urea, and fluorescein isothiocyanate-labeled dextran (FITC-dextran) of various average molecular weights (4, 20, 59–77 (mean = 68), 250, 500, and 2000 kDa), rhodamine 6G, rhodamine B, and horseradish peroxidase (HRP) were purchased from Merck Millipore (Burlington, MA, USA). Carboxylic acid-functionalized polystyrene (PS-COOH, 1.86 ± 0.03 µm) particles and green-fluorescent PS-COOH (1.20 ± 0.04 µm) particles were purchased from microParticles GmbH (Berlin, Germany). Ultrapure water (18.2 MΩ cm), obtained from a three-stage Millipore Milli-Q plus 185 purification system (Millipore Corporation, Burlington, MA, USA), was used for all experiments.

To determine the polydispersity index of the synthesized polymers, gel permeation chromatography (GPC) measurements (Shimadzu, Kyoto, Japan) were performed using poly(methyl methacrylate) standards. The column temperature was set at 50 °C, the flow rate was set at 1.0 mL min^{-1}, and DMF containing 50 mM LiBr was used as eluent. The chemical structures of the synthesized molecules and polymers, and molecular weights of the synthesized polymers were determined by proton nuclear magnetic resonance (^{1}H NMR) spectroscopy on a 500 MHz NMR spectrometer (Agilent Technologies, Santa Clara, CA, USA). Dynamic light scattering (DLS) size and ζ-potential measurements were performed on a Zetasizer NanoZS instrument (Malvern Instruments, Malvern, UK). Scanning electron microscopy (SEM) analysis was conducted on a FlexSEM microscope (Hitachi, Japan). Transmission electron microscopy (TEM) measurements were performed on an FEI Tecnai F20 microscope (FEI Company, Hillsboro, OR, USA)

operating at 200 kV to obtain TEM and high-angle annular dark-field (HAADF) images, and energy-dispersive X-ray spectroscopy (EDX) mapping data. Atomic force microscopy (AFM) experiments were conducted using a JPK NanoWizard II BioAFM (JPK Instruments AG, Berlin, Germany) with tapping-mode cantilevers. The mechanical properties of the capsules dispersed in water were determined using a Cypher atomic force microscope (Asylum Research, Goleta, CA, USA) with contact mode cantilevers. Differential interference contrast (DIC) microscopy and fluorescence microscopy images were taken on a Nikon A1R+ confocal laser scanning microscope (Nikon Corporation, Tokyo, Japan). HeLa and RAW 264.7 cells were purchased from the American Type Culture Collection (Manassas, VA, USA). HeLa and RAW 264.7 cells with passage numbers of 50–60 and 10–15, respectively, were used in this study and all cells passed the mycoplasma test. Dulbecco's phosphate-buffered saline (DPBS), Dulbecco's modified Eagle medium (DMEM), and 2,3-bis[2-methoxy-4-nitro-5-sulfophenyl]2*H*-tetrazolium-5-carboxyanilide inner salt (XTT) were obtained from Life Technologies (Carlsbad, CA, USA).

Synthesis of bis(α,α′-dimethyl-α′′-acetic acid)-trithiocarbonate (BDAT)

BDAT, a biscarboxyl acid-terminated trithiocarbonate chain transfer agent (CTA), was synthesized using a reported protocol[1] with slight modifications. Specifically, carbon disulfide (10.96 g, 8.66 mL), chloroform (43.00 g, 28.86 mL), acetone (20.92 g, 26.68 mL), and tetrabutylammonium hydrogen sulfate (0.96 g) were mixed with mineral spirits (48 mL) in a 500 mL round flask under nitrogen (or argon). Sodium hydroxide (40.32 g) was dissolved in water (80 mL) and added dropwise over 90 min to keep the temperature below 25 °C. The reaction was stirred overnight under nitrogen (or argon). Water (360 mL) was then added to dissolve the solid, followed by concentrated HCl (48 mL) (*caution! gas, mercaptan odor*) to acidify the aqueous layer. The mixture was stirred for 30 min with nitrogen (or argon) purge. The synthesized CTA was washed with deionized water (300 mL, 10000 rpm for 2 min, ×3) and then purified by recrystallization in acetone/hexane (4:1 (v/v), 5 × 2 mL) to obtain a yellow powder. The synthesized BDAT was kept at −20 °C for further use.

Synthesis of Biscarboxylic Acid-Functionalized Polymers

Biscarboxylic acid-functionalized poly(methyl acrylate) (PMA), poly(butyl acrylate) (PBA), polystyrene (PS), and poly(PEGA) (P(PEGA)) were synthesized by reversible addition–fragmentation chain transfer polymerization. To synthesize PMA, MA (3.05 g, 35.4 mmol) was dissolved in DMF (5 mL) containing AIBN (0.0058 g, 35.4 μmol) and BDAT (0.10 g, 354 μmol). To synthesize PBA, BA (18.2 g. 14.2 mmol) was dissolved in DMF (5 mL) containing AIBN (0.0116 g, 70.8 μmol) and BDAT (0.20 g, 708 μmol). To synthesize PS, styrene (6.5 g, 62.0 mmol) was dissolved in DMF (5 mL) containing AIBN (0.0020 g, 12.4 μmol) and BDAT (0.035 g, 124 μmol). To synthesize P(PEGA), PEGA (8.5 g, 17.7 mmol) was dissolved in DMF (5 mL) containing AIBN (0.0058 g, 35.4 μmol) and BDAT (0.10 g, 354 μmol). The solution was then degassed by argon purging for 30 min and then heated at 70 °C for 2 h (PMA and PBA), 96 h (PS), or 1 h (P(PEGA)). The polymerization was quenched by introducing oxygen. The synthesized PMA, PBA, PS, and P(PEGA) were precipitated by adding diethyl ether (200 mL) for PMA, hexane (200 mL) for PBA and P(PEGA), or methanol (200 mL) for PS, and the precipitated polymers were recovered by centrifugation (10000 *g*, 10 min). The same procedure was repeated three times, and the purified polymers were dried in vacuo and stored at −20 °C for further use.

Synthesis of Biscatechol-Functionalized Polymers

To synthesize biscatechol-functionalized polymers, the synthesized biscarboxylic acid-polymers were dissolved in DMF containing HATU and TEA and then degassed by purging with argon for 15 min. Dopamine·HCl was then added to the mixture, and the reaction solution was degassed by argon bubbling for 30 min. The molar ratio of biscarboxylic acid-polymer, dopamine·HCl, TEA, and HATU was 1:10:10:2.2. The reaction was allowed to proceed for 24 h at room temperature with stirring. After the reaction, the synthesized biscatechol-polymers were precipitated into diethyl ether (200 mL) for PMA, hexane (200 mL) for PBA and P(PEGA), or methanol (200 mL) for PS and washed twice with appropriate solvents depending on the polymer type. For further purification, dialysis (6000–8000 Da molecular weight cutoff, Thermo Fisher Scientific, USA) was performed for 2 days against acidic water (adjusted

to pH 4), followed by lyophilization to obtain purified biscatechol-polymers. The resulting polymers were stored at −20 °C for further use.

Synthesis of Metal–Phenolic Network (MPN) Capsules

PMA–Fe^{III}, PBA–Fe^{III}, PS–Fe^{III}, and P(PEGA)–Fe^{III} MPN capsules were synthesized using the same preparation method. A template particle dispersion, i.e., PS-COOH (1.86 ± 0.03 µm, 100 mg mL^{-1}) (50 µL), was transferred to a 1.7 mL microcentrifuge tube and washed twice with water. Specifically, the PS-COOH particles were washed with water by vortexing and sonication for 1–2 min and then pelleted by centrifugation (2000 *g*, 2 min). The supernatant was then discarded and the process was repeated. A biscatechol-polymer stock solution and $FeCl_3 \cdot 6H_2O$ solution were prepared in DMF. These solutions were added to the PS-COOH particle suspension after which water (380 µL) was added to obtain final concentrations of 10 mg mL^{-1} PS-COOH particles, 0.25–2.0 mM for polymer, and 0.5–4 mM for Fe^{III} ions, respectively, with vortexing for 2 min. The catechol/Fe^{III} ion ratio was maintained at 1:1 and the final DMF content was 24 % (v/v). To raise the pH above 7, MOPS buffer (25 mM, pH 7.4, 0.7 mL) was added, leading to the formation of bis- and tris-coordination complexes between biscatechol-polymers and Fe^{III} ions (DMF content = 10%). Excess and unreacted materials were then removed by pelleting the particles (2000 *g*, 2 min) and the supernatant was discarded. The MPN-coated particles were washed three times with water (500 µL) by repeated centrifugation (2000 *g*, 2 min) and redispersion. The particles were then resuspended in water (50 µL), and THF (1 mL) was added to remove the template particles. After 1 h, the MPN capsules were pelleted through centrifugation (2000 *g*, 2 min) and washed with THF (500 µL) five times. At the final THF washing step, the capsules were pelleted through centrifugation (2000 *g*, 3 min) and the supernatant was discarded. The resulting polymer–Fe^{III} MPN capsules were washed with DMF once and resuspended in DMF (500 µL). Hydrophobic polymer–metal ion MPN capsules composed of different metal ions (Al^{III}, Mn^{II}, Cr^{III}, and Cu^{II}), as well as hydrophobic polymer/$P(PEGA)_{58}$–Fe^{III} MPN capsules were prepared using the same preparation method as described above. The molar ratio of the

hydrophobic polymer/P(PEGA)$_{58}$ was 1:1 and the concentration of the polymers in the assembly solution was 0.5 mM.

Molecular Dynamics (MD) Simulations

All-atom MD simulations were performed to elucidate the dynamics among 9 biscatechol-PMA or 9 biscatechol-PBA in water/DMF mixture (DMF content = 24%), with a total system density of 0.05 g cm^{-3}. Each biscatechol-PMA and biscatechol-PBA chain consisted of 100 MA and 118 BA monomers, respectively, corresponding to molecular weights of 8900 and 15 400 g mol^{-1}, respectively. To elucidate the interactions between a solid substrate and the biscatechol-PMA or biscatechol-PBA films, 10 PS chains were used as the substrate. The substrate was kept frozen and interacted with the polymer films only via van der Waals and electrostatic interactions.

The initial configurations were generated using the Materials and Processes Simulations (MAPS) 4.3 platform.[2] All systems were simulated in the NVT (constant number of atoms, volume, and temperature) ensemble at 298.15 K for at least 1 ns using the velocity–Verlet algorithm[3] with a timestep of 1 fs, applying periodic boundary conditions. The interatomic interactions were modeled based on the transferable, extensible, accurate and modular force field (TEAM-FF). Partial charges were assigned to each atom based on the bond increment method. The particle–particle particle–mesh solver was used to calculate the long-range Coulombic interactions. Simulations were performed on the large-scale atomic/molecular massively parallel simulator (LAMMPS) code[4] using the University of Melbourne's High-Performance Computing cluster.

Preparation of Amine-Terminated Glass Substrates

For the modification of glass substrates, a 0.5% 3-(aminopropyl)triethoxysilane (APTES) solution was first prepared by dissolving APTES (400 μL) in ethanol (80 mL). The glass substrates were then immersed in 0.5% APTES solution for 24 h, followed by thorough rinsing with ethanol and water. The obtained amine-functionalized glass substrates were dried under a stream of air and kept dry until further use.

Structural Stability Evaluation of MPN Capsules

A dispersion (5 µL) of PMA_{100}–Fe^{III} MPN capsules was added to an APTES-modified glass substrate and left standing for 30 min for the capsules to attach to the base of the glass substrate. Water (40 µL) was then added to the capsule area on the glass substrate to examine the initial states of the capsules in water. To investigate the stability of the PMA_{100}–Fe^{III} MPN capsules under various conditions, water was replaced with 0.5 M HCl, 0.5 M NaOH, 100 mM EDTA, 100 mM NaCl, 100 mM Tween 20, 100 mM urea, 50 mM MOPS (pH 8), DMF, or DMSO. After 10, 30, and 60 min, DIC microscopy images were captured.

Mechanical Studies

A fresh PMA_{100}–Fe^{III} or PBA_{118}–Fe^{III} MPN capsule solution (5 µL) was dropped onto a sectioned amine-terminated glass substrate (1 cm × 1 cm) and left undisturbed for 30 min. Water (200 µL) was then added to the capsule area on the glass substrate. The measurements were performed using a Cypher atomic force microscope (Asylum Research, Goleta, CA, USA) in ultrapure water (18.2 MΩ cm) with BioLever mini cantilevers (spring constant 0.02–0.14 N m^{-1}, Olympus, Tokyo, Japan). The spring constants of the cantilevers were determined to be 0.070–0.111 N m^{-1}. The location of individual capsules was identified through AFM imaging under tapping mode. An individual capsule was then indented with the AFM tip at a specified applied force (1.5 nN) and a constant velocity (0.5 µm s^{-1}) under contact mode, and the resulting force–deformation (F–δ) curve was recorded. The F–δ curves of five different capsules were collected and analyzed using Asylum Research software.

Permeability Studies

To analyze the permeability of the MPN capsules, the capsules were attached onto an amine-terminated glass substrate following the same protocol as that used for the DIC microscopy analyses. A prepared PMA_{100}–Fe^{III} or PBA_{118}–Fe^{III} MPN capsule solution (5 µL) was added to the amine-functionalized glass substrate and left undisturbed for 30 min for the capsules to attach to the base of the glass substrate. Water

(40 µL) was then added to the capsule area on the glass substrate, followed by FITC-dextran (1 mg mL^{-1}; M_w 4, 20, 68, 250, 500, and 2000 kDa). After 5 min, confocal laser scanning microscopy (CLSM) images of the capsules were taken. Capsules with dark interiors were considered to be impermeable, whereas capsules with interiors of fluorescence intensity similar to the outer environment were considered to be permeable. Approximately 50–100 capsules were examined. The permeability of the capsules is reported as percentage of permeable capsules.

Loading of Fluorescent Molecules and Proteins into MPN Capsules

To load fluorescent molecules (e.g., 2000 kDa FITC-dextran, rhodamine 6G, and rhodamine B) into PMA_{100}–Fe^{III}, PBA_{118}–Fe^{III}, or $P(PEGA)_{58}$–Fe^{III} MPN capsules, MPN capsules were attached to an amine-terminated glass substrate following the same protocol as that used for the structural stability studies. Briefly, an MPN capsule solution (5 µL) was added to the APTES-modified glass substrate and then, after 30 min, water (40 µL) was added to the capsule area. Thereafter, 5 µL of dye solution (i.e., 2000 kDa FITC-dextran (10 mg mL^{-1}), rhodamine B (1 mM), or rhodamine 6G (1 mM)) was added to the capsule area on the glass substrate. After incubation for 1–60 min, CLSM images of the capsules were taken.

For protein loading into the MPN capsules, 1 mM PBA_{118}–Fe^{III} capsules (in 450 µL DMF) were incubated with HRP solution (50 µL, 10 mg mL^{-1}) for 4 h under shaking at 1200 rpm. The HRP-loaded capsules were then washed with water/DMF (1:9 v/v) (500 µL; 2000 *g*, 2 min) twice to remove unbound HRP, and then resuspended in water (450 µL). The loading was calculated to be 36% based on the absorbance at 280 nm.[5]

HRP Activity Studies

A colorimetric assay was used to characterize and compare the activities of free HRP and HRP-loaded PBA_{118}–Fe^{III} capsules. HRP can catalyze the oxidation of amplex red by H_2O_2 to resorufin; this reaction can be monitored by the strong absorbance at 560 nm owing to the formation of resorufin.[6] For the

activity study, H_2O_2 (50 μL, 20 mM) and amplex red (2.5 μL, 1 mg mL^{-1}) were added to PBS (10 mM, pH 7.4), followed by the addition of HRP or HRP-loaded PBA$_{118}$–Fe^{III} capsules to reach a final HRP concentration of 0.1 μg mL^{-1}. The final volume of the reaction solution was 500 μL. The activity of HRP (through the formation of resorufin) was studied by monitoring the absorbance at 560 nm in 10 s intervals by UV–vis spectrophotometry.

Synthesis of MPN Particles

Fluorescent MPN particles for conducting cellular uptake experiments were prepared as follows. PS-COOH particles displaying green fluorescence (1.20 ± 0.04 µm) were washed with water twice by vortexing and sonication for 1–2 min and then pelleted by centrifugation (2000 *g*, 2 min). Solutions of biscatechol-PMA and biscatechol-P(PEGA) at different molar ratios (0:1, 1:4, and 1:2) and Fe^{III} ions were then added to the PS-COOH particles and mixed with vortexing to obtain final concentrations of 2.5 mg mL^{-1} particles, 0.5 mM polymer, and 1 mM Fe^{III} ions, respectively, in water/DMF mixture (DMF content = 24 % (v/v)). To form bis- and tris-coordination complexes, MOPS buffer (25 mM, pH 7.4, 0.7 mL) was added. The resulting particles were then washed with water three times (500 μL, 2000 *g*, 2 min) and resuspended in water (1.5 mL) water. For the cytotoxicity studies, nonfluorescent PS-COOH particles (1.86 ± 0.03 µm) were used for the preparation of MPN particles.

Cellular Uptake Properties of MPN Particles

The intracellular binding properties of the fluorescent MPN particles (i.e., P(PEGA)$_{58}$–Fe^{III} MPN, PMA$_{100}$/P(PEGA)$_{58}$(1:4)–Fe^{III} MPN, and PMA$_{100}$/P(PEGA)$_{58}$(1:2)–Fe^{III} MPN particles) were investigated by flow cytometry and CLSM. For flow cytometry, HeLa cells were seeded in a 24-well plate at 5 × 10^4 cells per well and then cultured in DMEM with 10% fetal bovine serum (FBS) at 37 °C with 5% CO_2 for 20 h to facilitate cellular adhesion on the plates. The MPN particles were then added to the cells to achieve a final cell-to-particle ratio of 1:10 and incubated for 4 h to examine the cell association behavior. After incubation, the supernatant was discarded, and the cells were washed with

DPBS (500 µL) three times. Trypsin solution (1×, 200 µL) was added to facilitate cell detachment, and the solution was maintained in an incubator (37 °C with 5% CO_2) for 5 min. For neutralization, DMEM (300 µL) was added and the detached cells were washed with DPBS (1 mL) three times through centrifugation (350 *g*, 5 min, 4 °C). The cells were dispersed in DPBS (800 µL) and cell association was analyzed by flow cytometry. The relative fluorescence intensity (RFI) data refer to the fluorescence intensity of treated cells relative to the fluorescence intensity of untreated cells.

For CLSM analysis, HeLa cells were seeded into 8-well Lab-Tek chambered coverglass slides (Thermo Fisher Scientific, Waltham, MA, USA) at a cell density of 4×10^4 cells per well and then cultured in DMEM with 10% FBS at 37 °C with 5% CO_2 for 20 h to allow cellular adhesion on the plates. The MPN particles were incubated for 4 h while keeping the cell-to-particle ratio as 1:10. After incubation, the supernatant was discarded and the cells were washed with DPBS (400 µL) twice. Cells were fixed by adding 4% paraformaldehyde (200 µL) for 20 min at room temperature, then washed with DPBS (400 µL) twice. The cell membranes were stained with wheat germ agglutinin–Alexa Fluor 594 conjugate for 5 min on ice, and the cell nuclei were dyed with Hoechst 33342 for 10 min at room temperature. CLSM imaging was performed to monitor the cellular uptake of the particles on a microscope equipped with a Plan Apo λ 60× 1.4 NA oil immersion objective, and 405, 488, and 561 nm lasers, and 450/50, 525/50, and 595/50 bandpass emission filters. The images were processed using Fiji software.

Cytotoxicity Studies of Binary MPN Particles

For the cytotoxicity assessment, nonfluorescent PS-COOH particles (1.86 µm) were used for the preparation of binary MPN particles to avoid absorbance overlap at 475 nm that is observed between green-fluorescent particles used for the cell association experiments and the XTT method. HeLa and RAW 264.7 cells were seeded on a 96-well plate at a density of 8000 cells per well and incubated overnight. The culture media was then removed and replaced with either fresh media (for the cell control) or fresh media containing PS-COOH (control), $P(PEGA)_{58}$–Fe^{III} MPN, $PMA_{100}/P(PEGA)_{58}(1{:}4)$–$Fe^{III}$ MPN, or $PMA_{100}/P(PEGA)_{58}(1{:}2)$–$Fe^{III}$ MPN particles at a cell-to-particle ratio of 1:25 or 1:100 for 24

or 48 h. The XTT assay was performed to examine the cytotoxicity of these binary MPN particles. The media was replaced with fresh media containing activated XTT (9 mL of 0.2 mg mL^{-1} XTT in DMEM with 10% FBS was activated by adding 22.5 μL of 0.6 mg mL^{-1} phenazine methosulfate in DPBS), and cells were further incubated for 3 h. Finally, the cells were screened on an Infinite M200 microplate reader (Tecan, Switzerland). Absorbance readings were measured at 475 nm with 30 s shaking, and a reference wavelength of 675 nm was used. Cell viability is expressed as a percentage by normalizing the absorbance to that obtained for untreated cells. All experiments were performed in triplicate, and data are presented as the mean ± standard deviation.

Minimum Information Reporting in Bio-Nano Experimental Literature (MIRIBEL)

The studies conducted herein, including material characterization, biological characterization, and experimental details, conform to the MIRIBEL reporting standard for bio–nano research,[7] and we include a companion checklist of these parameters herein.

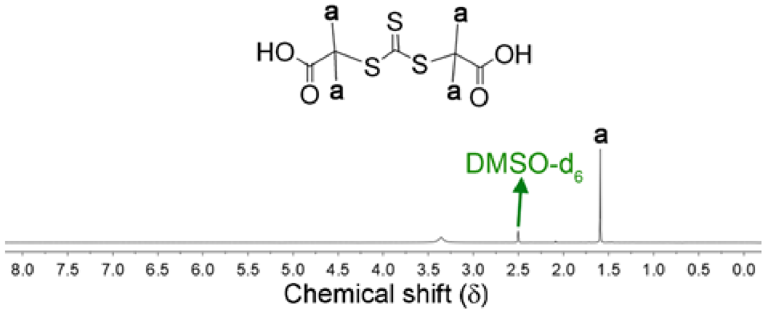

Figure S1. ^{1}H NMR (DMSO-d_6, 500 MHz) spectrum and chemical structure of BDAT.

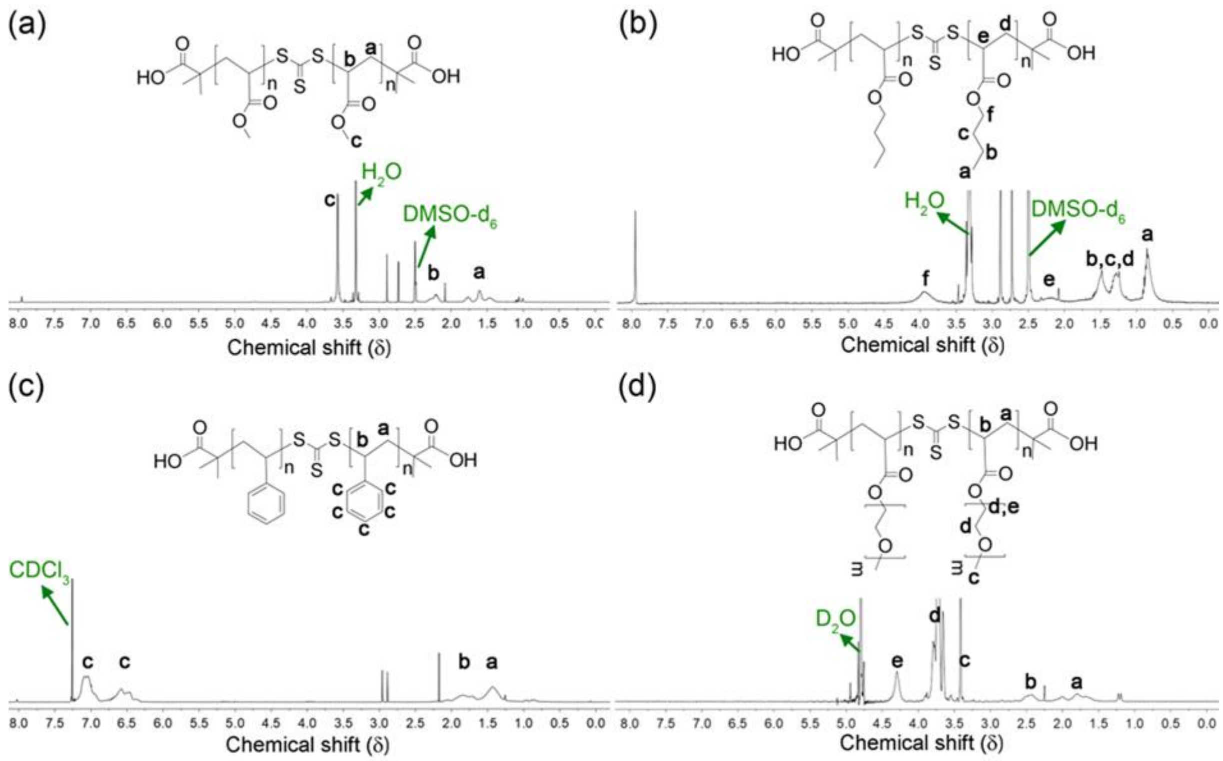

Figure S2. 500 MHz ^{1}H NMR spectra and chemical structures of (a) biscarboxylic acid-PMA_{100} (DMSO-d_6), (b) biscarboxylic acid-PBA_{118} (DMSO-d_6), (c) biscarboxylic acid-PS_{94} ($CDCl_3$), and (d) biscarboxylic acid-$P(PEGA)_{58}$ (D_2O).

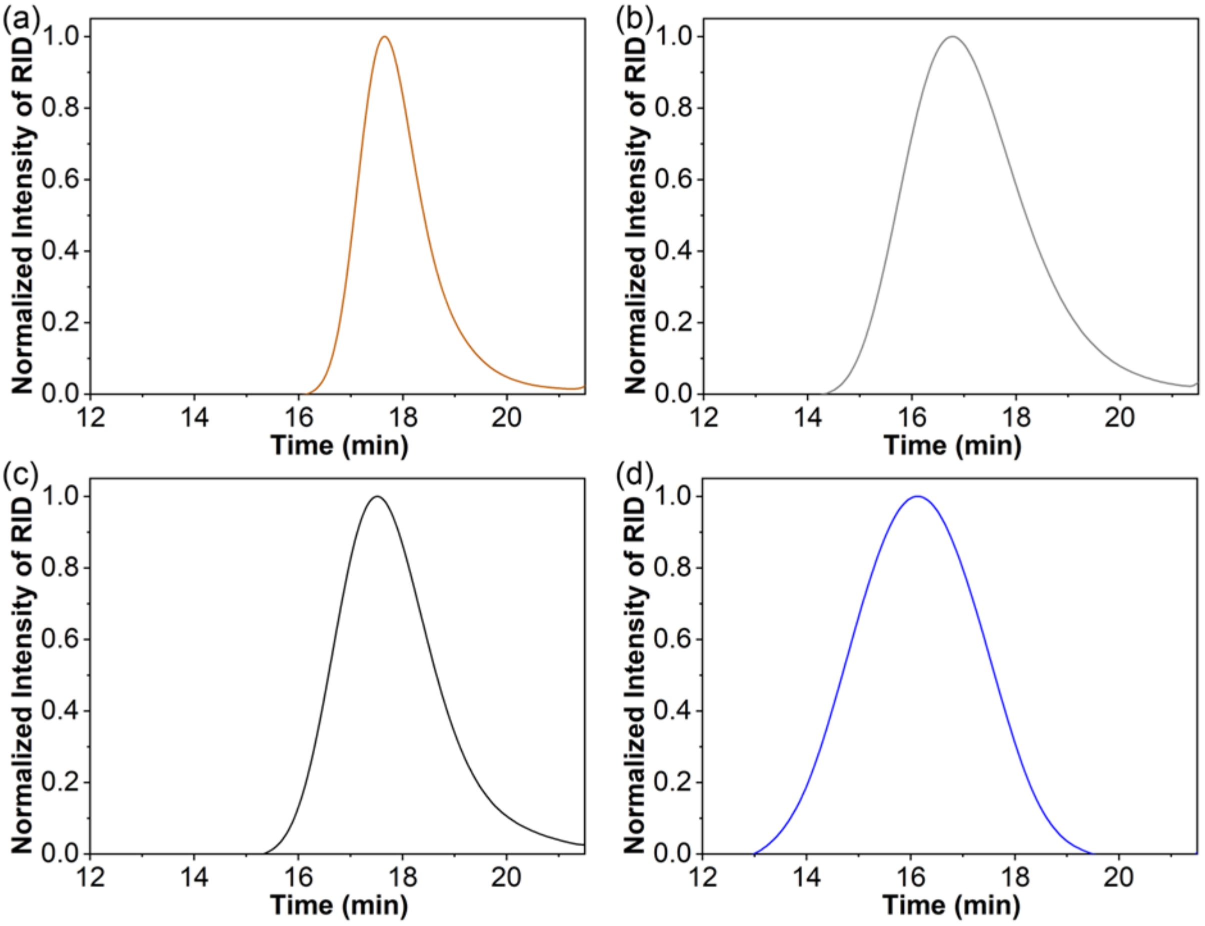

Figure S3. GPC traces (refractive index detector, RID) of (a) biscarboxylic acid-PMA_{100}, (b) biscarboxylic acid-PBA_{118}, (c) biscarboxylic acid-PS_{94}, and (d) biscarboxylic acid-P(PEGA)$_{58}$.

Table S1. Number- and weight-average molecular weight (M_n and M_w) of synthesized biscarboxylic acid-polymers[a]

Polymer	M_n (g mol^{-1})	M_w/M_n
Biscarboxylic acid-PMA_{100}	8900	1.16
Biscarboxylic acid-PBA_{118}	15400	1.35
Biscarboxylic acid-PS_{94}	10100	1.24
Biscarboxylic acid-P(PEGA)$_{58}$	28300	1.53

[a]M_n and dispersity (i.e., M_w/M_n) were determined by GPC using poly(methyl methacrylate) standards.

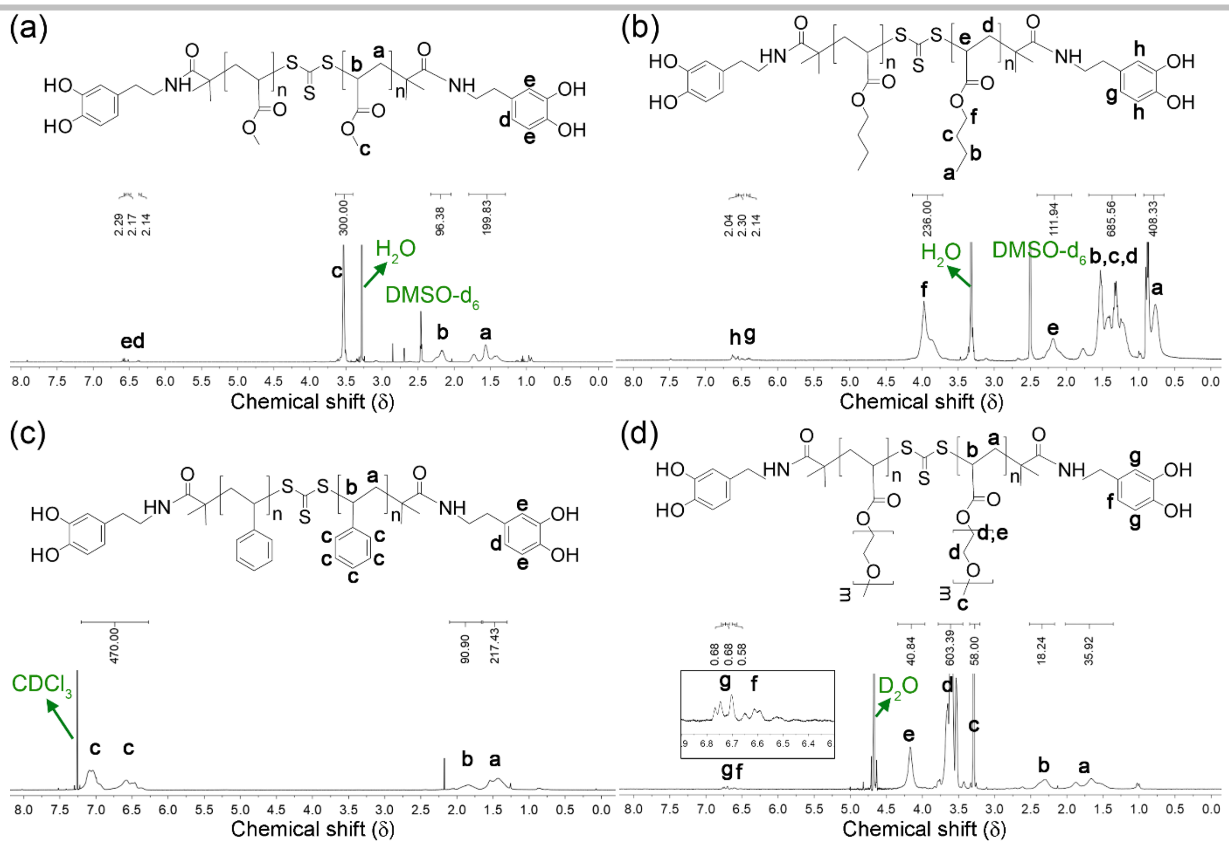

Figure S4. 500 MHz ^{1}H NMR spectra and chemical structures of (a) biscatechol-PMA100 (DMSO-d_6), (b) biscatechol-PBA118 (DMSO-d_6), (c) biscatechol-PS94 ($CDCl_3$), and (d) biscatechol-P(PEGA)58 (D_2O).

Table S2. Coupling yields of biscatechol-PMA100 and biscatechol-PBA118 based on ^{1}H NMR spectra[a]

Polymer	*from peak d*	*from peak e1*[b]	*from peak e2*[b]	Average coupling yield
Biscatechol-PMA100	107%	109%	115%	110%
Biscatechol-PBA118	107%	115%	102%	108%

[a]Biscatechol-PMA100 and biscatechol-PBA118 were selected to calculate the coupling yields as their molecular weights estimated from GPC (using poly(methyl methacrylate) standards) are considered a fair approximation to their actual molecular weights, in view of the similarity between the polymer structures.

[b]Peak e1 is located upfield (i.e., lower ppm) compared with peak e2.

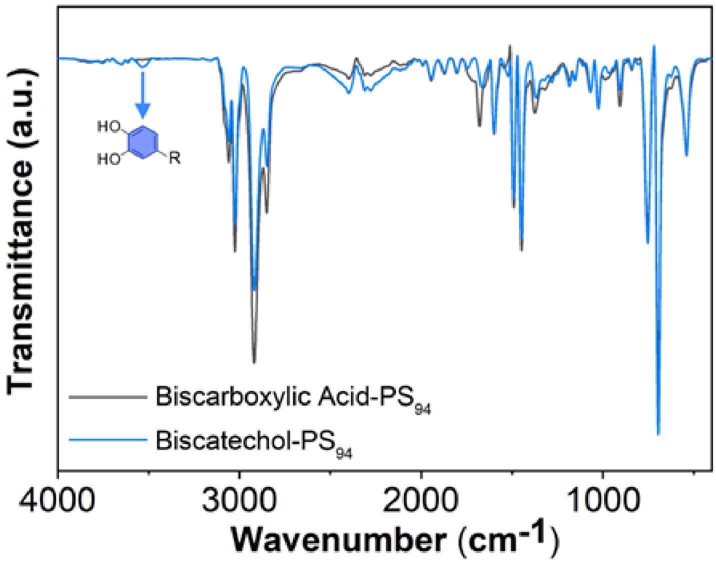

Figure S5. Fourier transform infrared spectra of biscarboxylic acid-PS_{94} and biscatechol-PS_{94}.

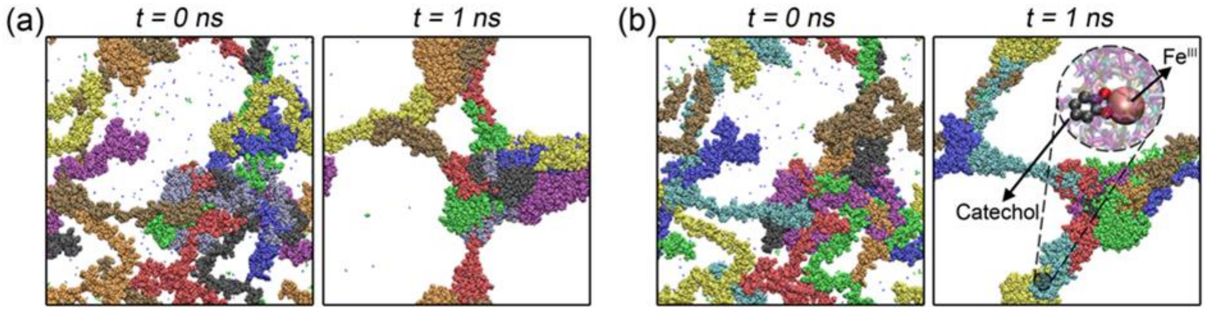

Figure S6. MD simulation snapshots of (a) PBA chains and (b) PBA–Fe^{III} complexes in water/DMF mixture (DMF content = 24%) at t = 0 and 1 ns. Polymer chains are color-coded.

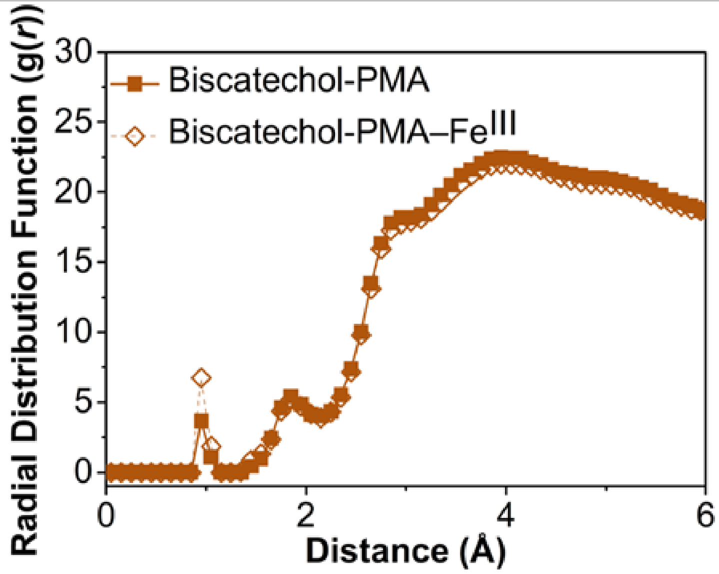

Figure S7. Radial distribution function, g(*r*), between water molecules and biscatechol-PMA or biscatechol-PMA–Fe^{III}.

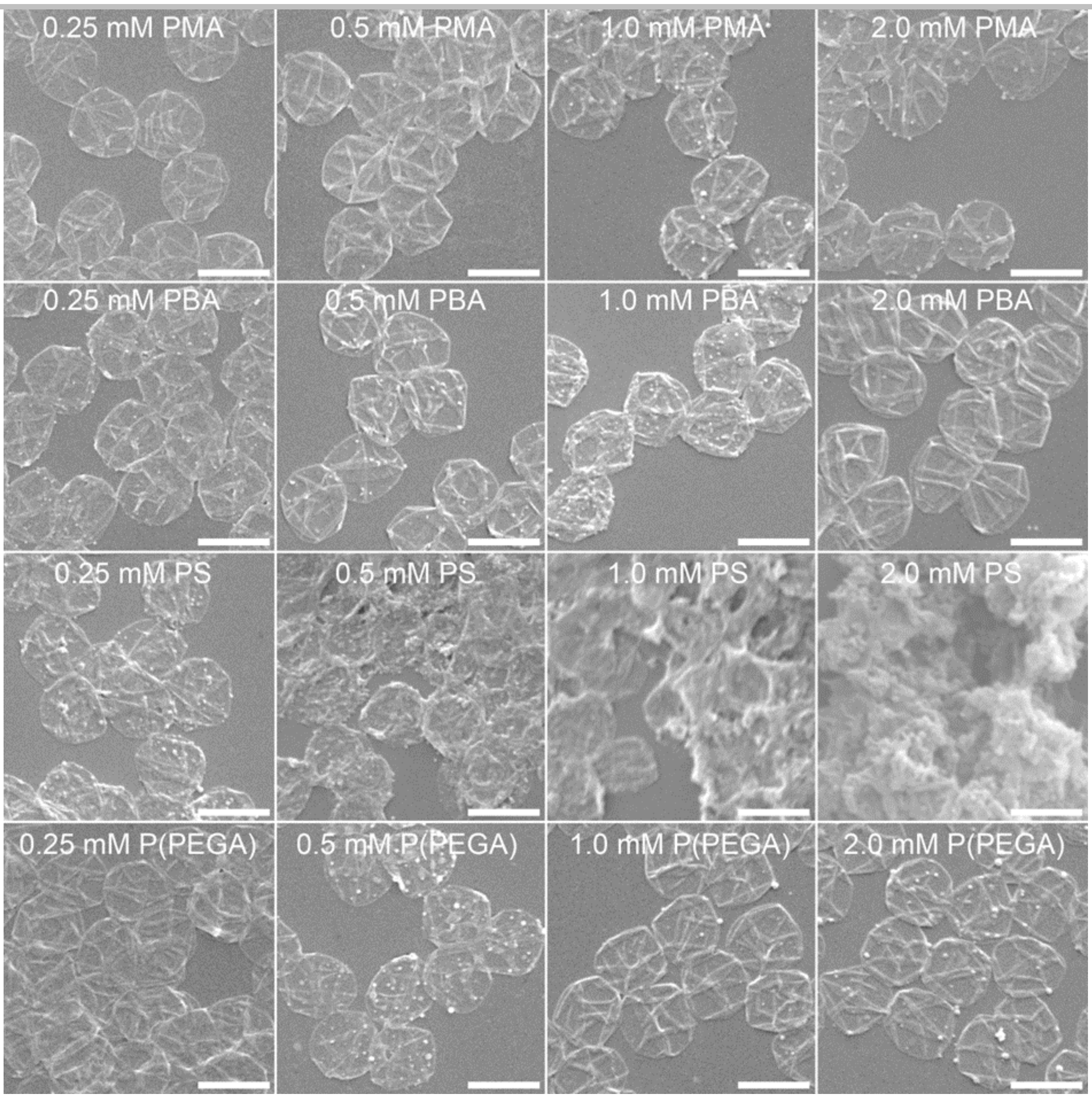

Figure S8. SEM images of PMA_{100}–Fe^{III}, PBA_{118}–Fe^{III}, PS_{94}–Fe^{III}, and $P(PEGA)_{58}$–Fe^{III} MPN capsules prepared using different polymer concentrations in the assembly solution and a constant catechol/Fe^{III} ion ratio of 1:1. Scale bars are 2 μm.

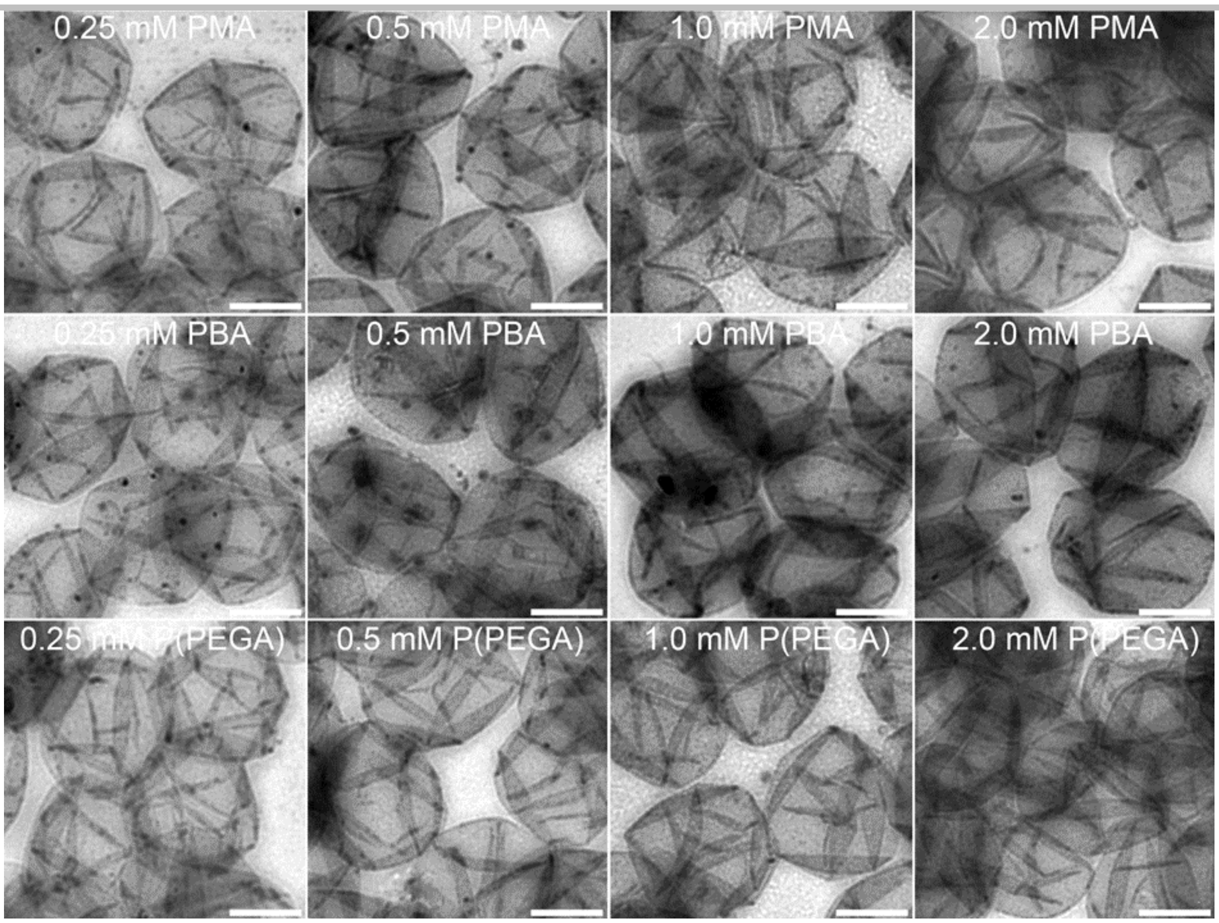

Figure S9. TEM images of PMA_{100}–Fe^{III}, PBA_{118}–Fe^{III}, and $P(PEGA)_{58}$–Fe^{III} MPN capsules prepared using different polymer concentrations in the assembly solution and a constant catechol/Fe^{III} ion ratio of 1:1. Scale bars are 1 μm.

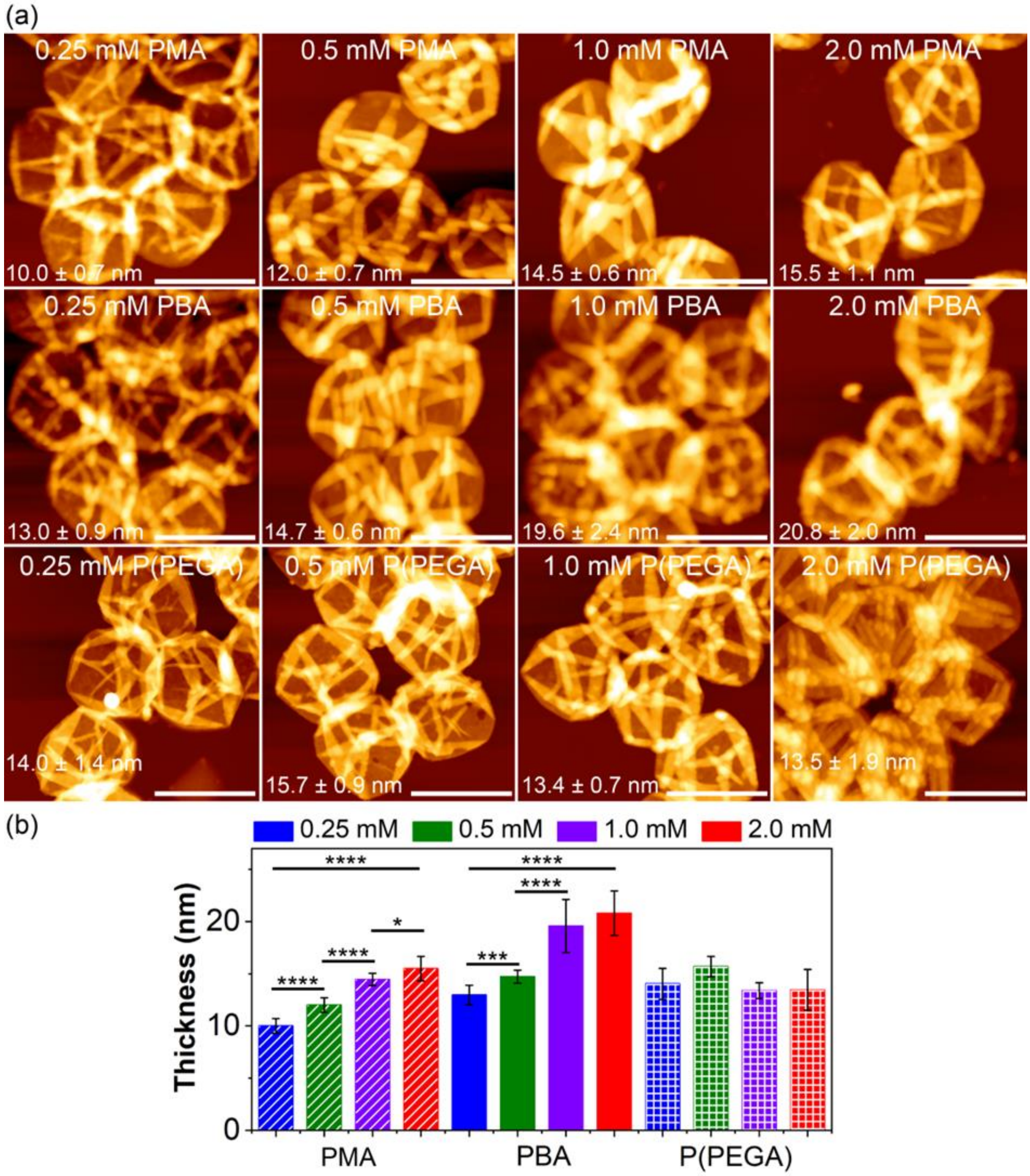

Figure S10. (a) AFM images of PMA_{100}–Fe^{III}, PBA_{118}–Fe^{III}, and $P(PEGA)_{58}$–Fe^{III} MPN capsules prepared using different polymer concentrations in the assembly solution and a constant catechol/Fe^{III} ion ratio of 1:1. Scale bars are 2 μm. (b) Shell thickness of the synthesized MPN capsules. Shell thickness was determined from AFM height–distance graphs and shown as the mean ± standard deviation of 10 independent AFM measurements for each capsule type. Statistical significance was determined by one-way analysis of variance (ANOVA): **** $p < 0.0001$, *** $p < 0.001$, and * $p < 0.05$.

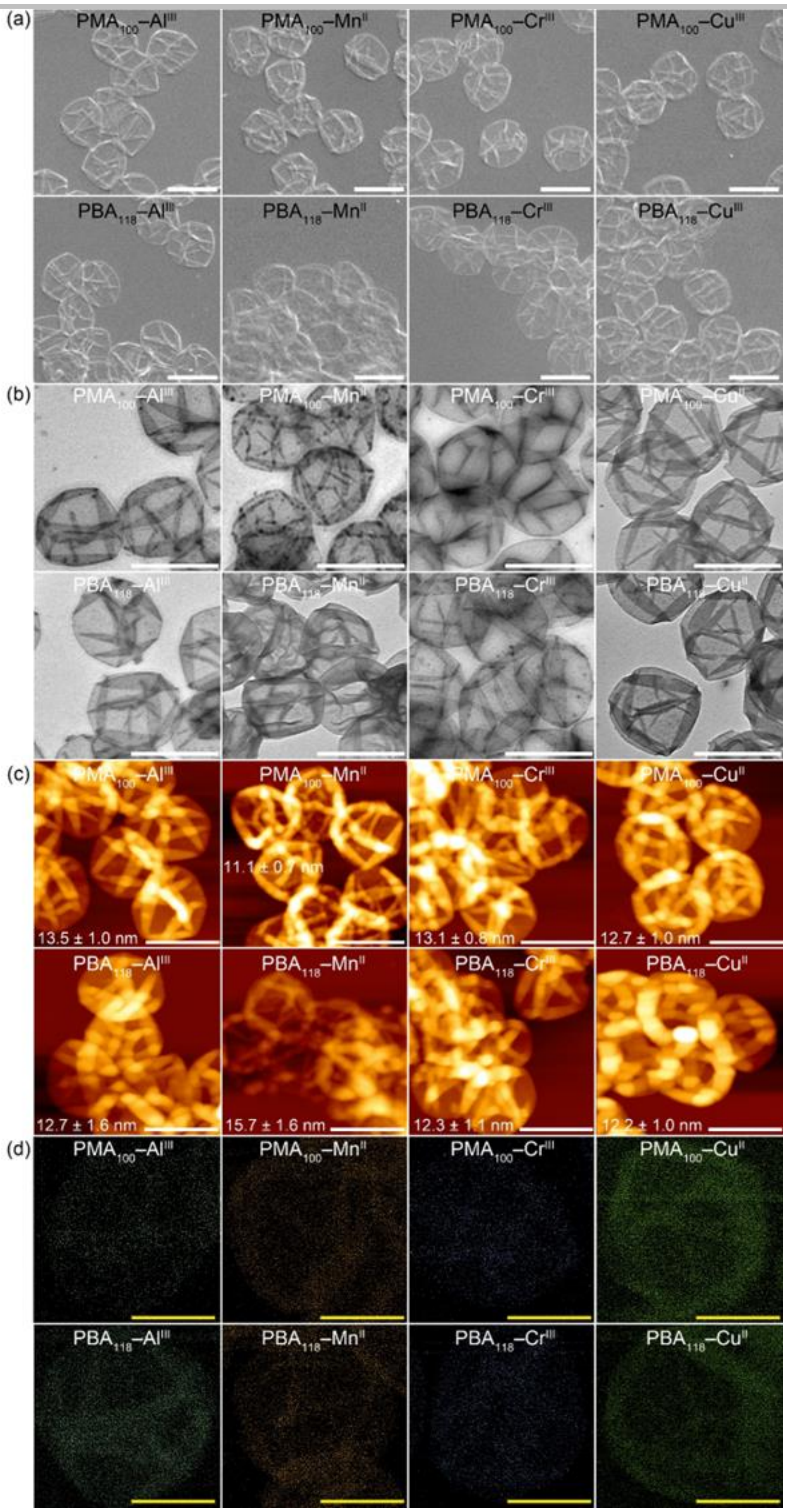
(a)
PMA100–AlIII
PMA100–MnII
PMA100–CrIII
PMA100–CuIII
PBA118–AlIII
PBA118–MnII
PBA118–CrIII
PBA118–CuIII
(b)
PMA100–AlIII
PMA100–MnII
PMA100–CrIII
PMA100–CuII
PBA118–AlIII
PBA118–MnII
PBA118–CrIII
PBA118–CuII
(c)
PMA100–AlIII
PMA100–MnII
PMA100–CrIII
PMA100–CuII
13.5 ± 1.0 nm
11.1 ± 0.7 nm
13.1 ± 0.8 nm
12.7 ± 1.0 nm
PBA118–AlIII
PBA118–MnII
PBA118–CrIII
PBA118–CuII
12.7 ± 1.6 nm
15.7 ± 1.6 nm
12.3 ± 1.1 nm
12.2 ± 1.0 nm
(d)
PMA100–AlIII
PMA100–MnII
PMA100–CrIII
PMA100–CuII
PBA118–AlIII
PBA118–MnII
PBA118–CrIII
PBA118–CuII

Figure S11. (a) SEM, (b) TEM, (c) AFM, and (d) EDX elemental mapping images of 0.5 mM PMA_{100}–metal ion and 0.5 mM PBA_{118}–metal ion MPN capsules. Scale bars are 2 μm (a–c) and 1 μm (d). Shell thickness was determined from AFM height–distance graphs and shown as the mean ± standard deviation of 10 independent AFM measurements for each capsule type.

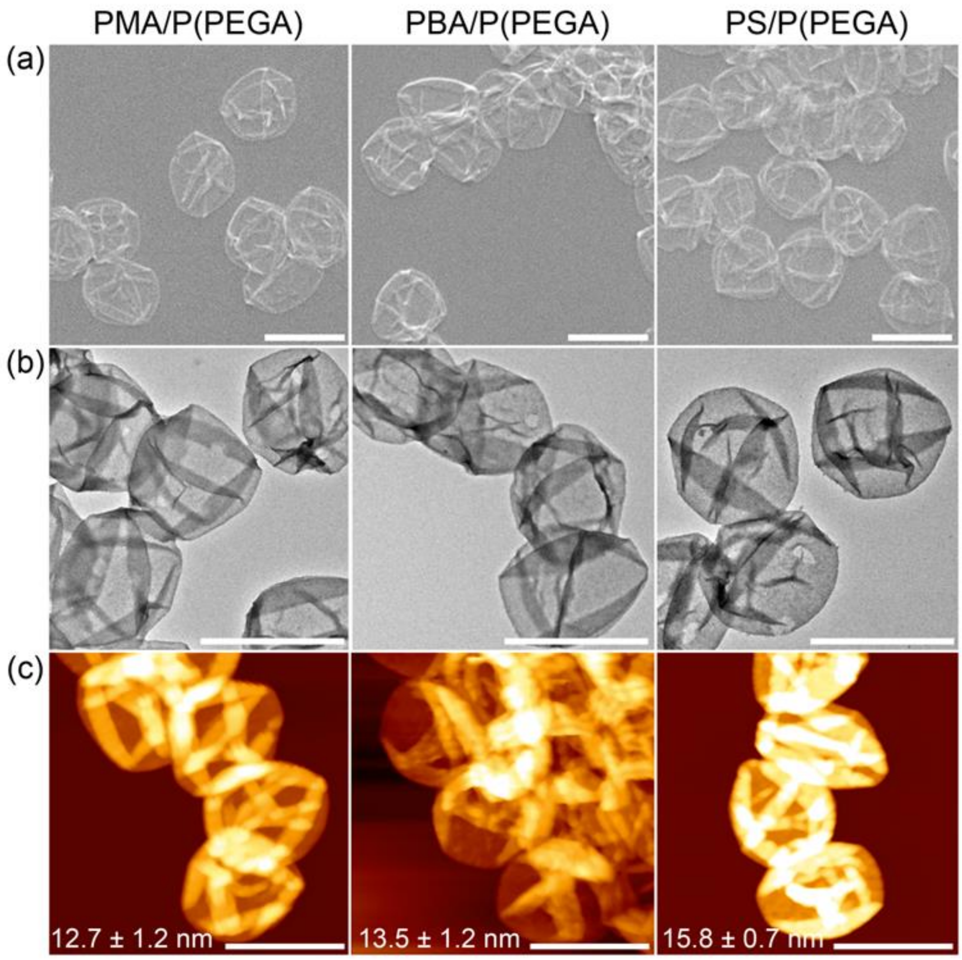

Figure S12. (a) SEM, (b) TEM, and (c) AFM images of 0.5 mM PMA_{100}/$P(PEGA)_{58}$(1:1)–Fe^{III}, PBA_{118}/$P(PEGA)_{58}$(1:1)–Fe^{III}, and PS_{94}/$P(PEGA)_{58}$(1:1)–Fe^{III} MPN capsules. Scale bars are 2 μm. Shell thickness was determined from AFM height–distance graphs and shown as the mean ± standard deviation of 10 independent AFM measurements for each capsule type.

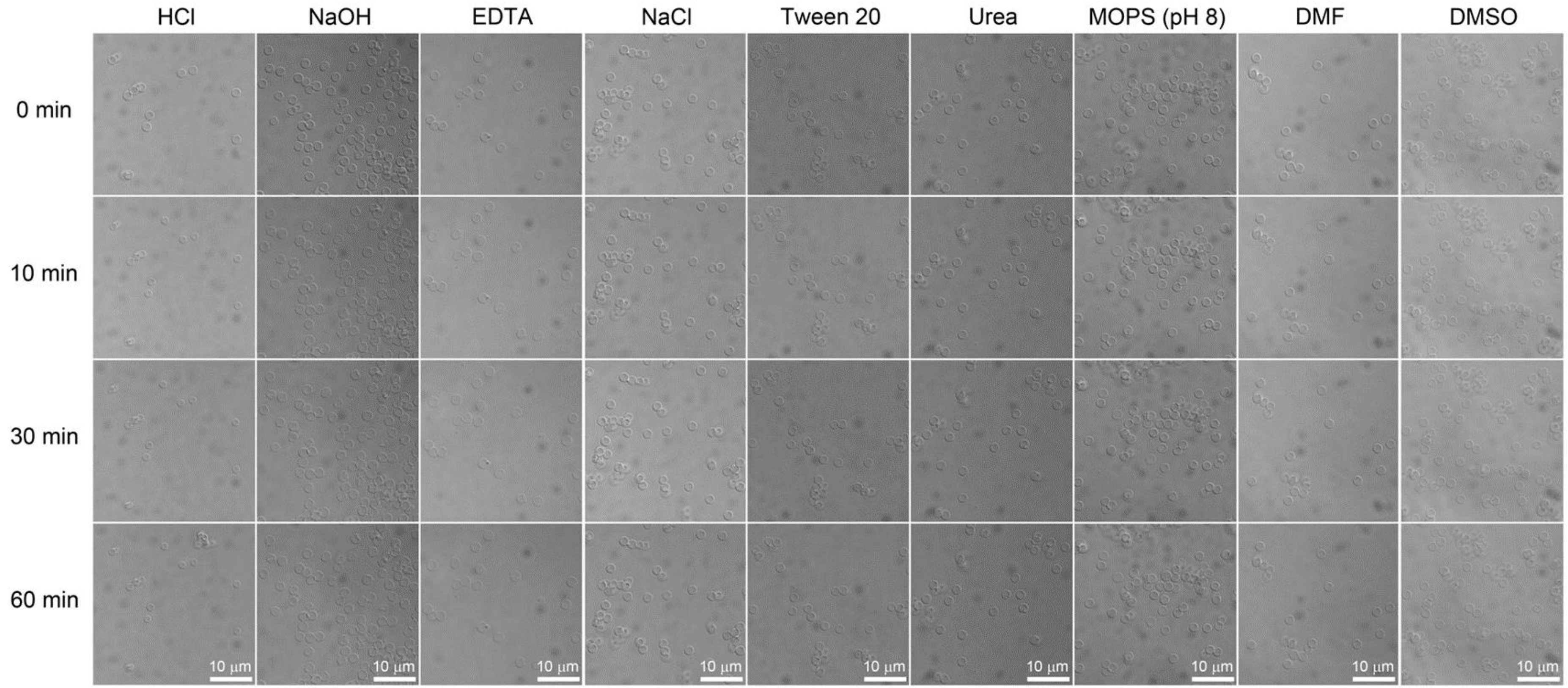

Figure S13. Stability of PMA_{100}–Fe^{III} MPN capsules in different environments. Representative DIC images of capsules after incubation for 0–60 min in water (control), 0.5 M HCl, 0.5 M NaOH, 100 mM EDTA, 100 mM NaCl, 100 mM Tween 20, 100 mM urea, 50 mM MOPS (pH 8), DMF, or DMSO.

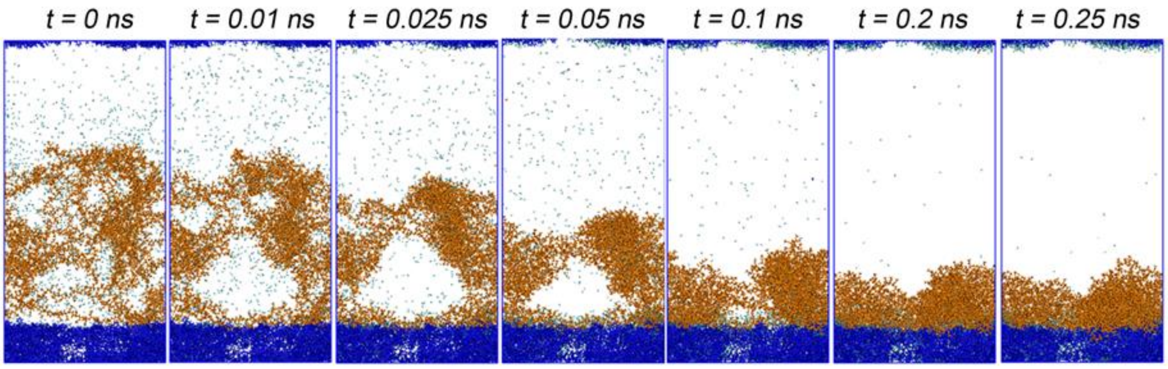

Figure S14. MD simulation snapshots of a biscatechol-PMA–Fe^{III} film (6 strands of PMA and 3 Fe^{III} ions) depositing on a PS substrate.

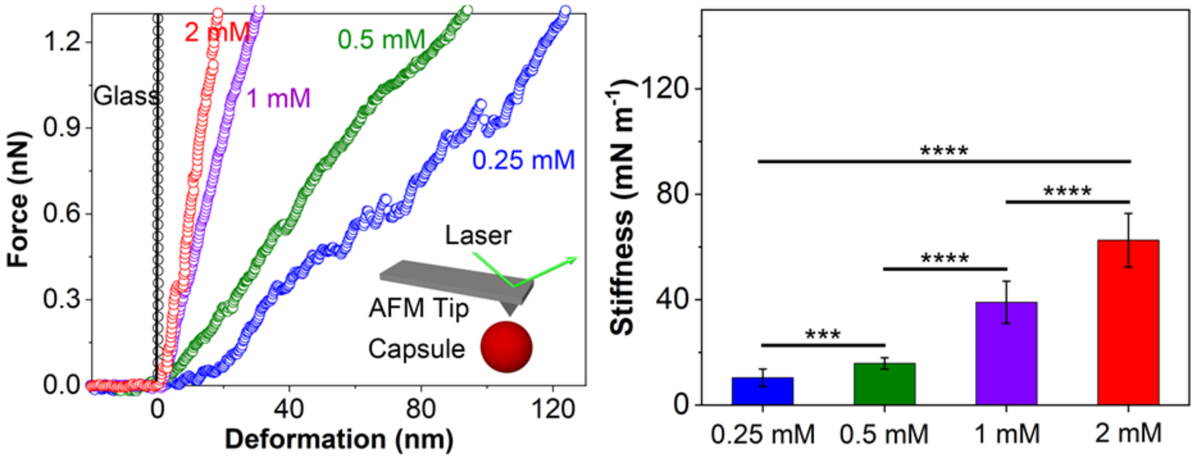

Figure S15. Representative *F*–*δ* curves and corresponding stiffness profiles of PMA_{100}–Fe^{III} MPN capsules prepared using different PMA concentrations (0.25–2.0 mM) and a constant catechol/Fe^{III} ion ratio of 1:1. The *F*–*δ* curve of a glass substrate is also shown for comparison. The data are shown as mean stiffness ± standard deviation (n = 10). Statistical significance was determined by one-way ANOVA: **** $p < 0.0001$ and *** $p < 0.001$.

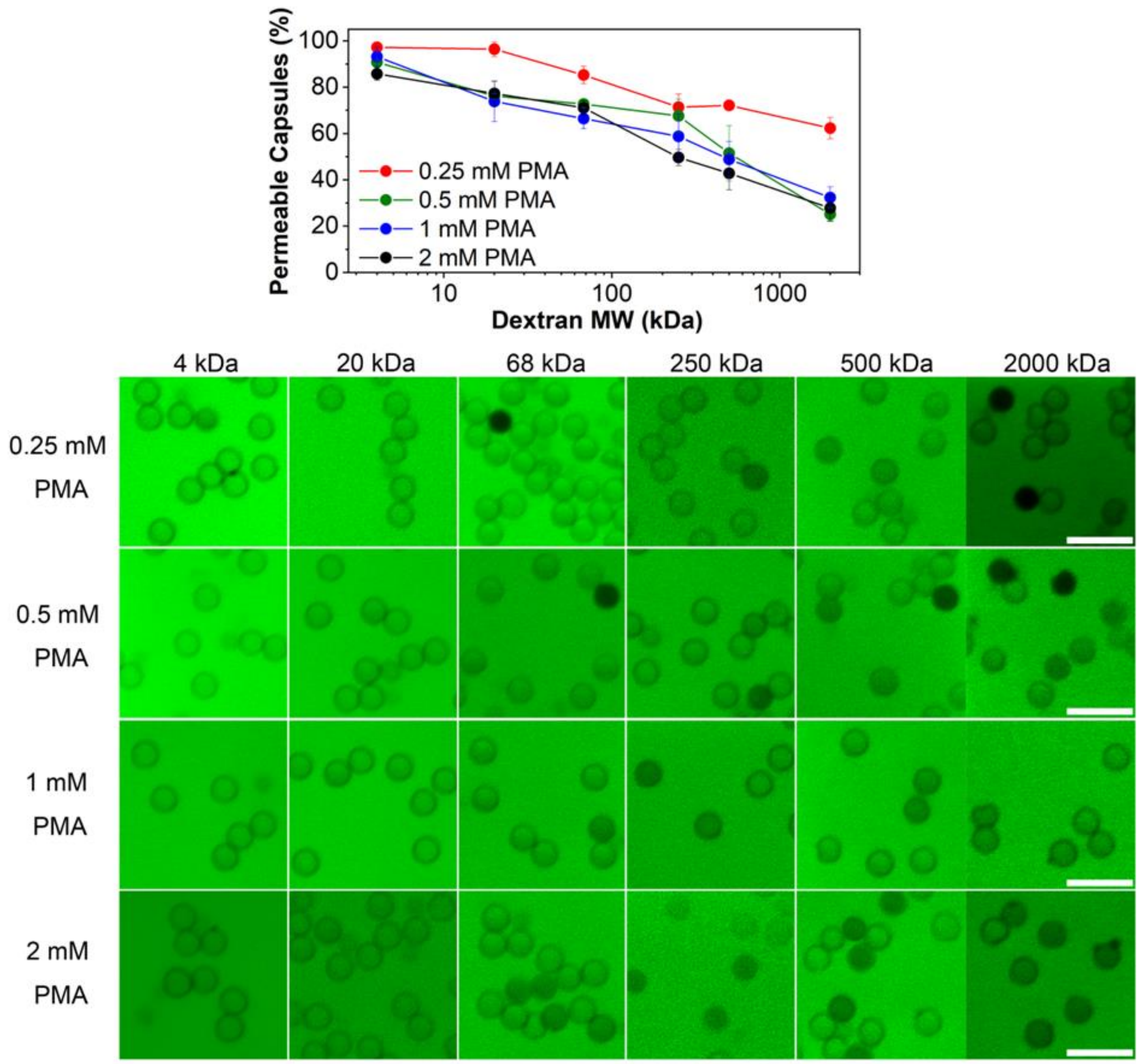

Figure S16. Comparison of the permeability of PMA_{100}–Fe^{III} MPN capsules to FITC-dextran of M_w ranging from 4 to 2000 kDa (above) and corresponding representative confocal microscopy images (below; scale bars are 5 μm). The PMA_{100}–Fe^{III} MPN capsules were prepared using different PMA concentrations in the assembly solution and a constant catechol/Fe^{III} ion ratio of as 1:1. The permeability data are shown as the mean ± standard deviation of three independent experiments; 50–100 capsules were examined.

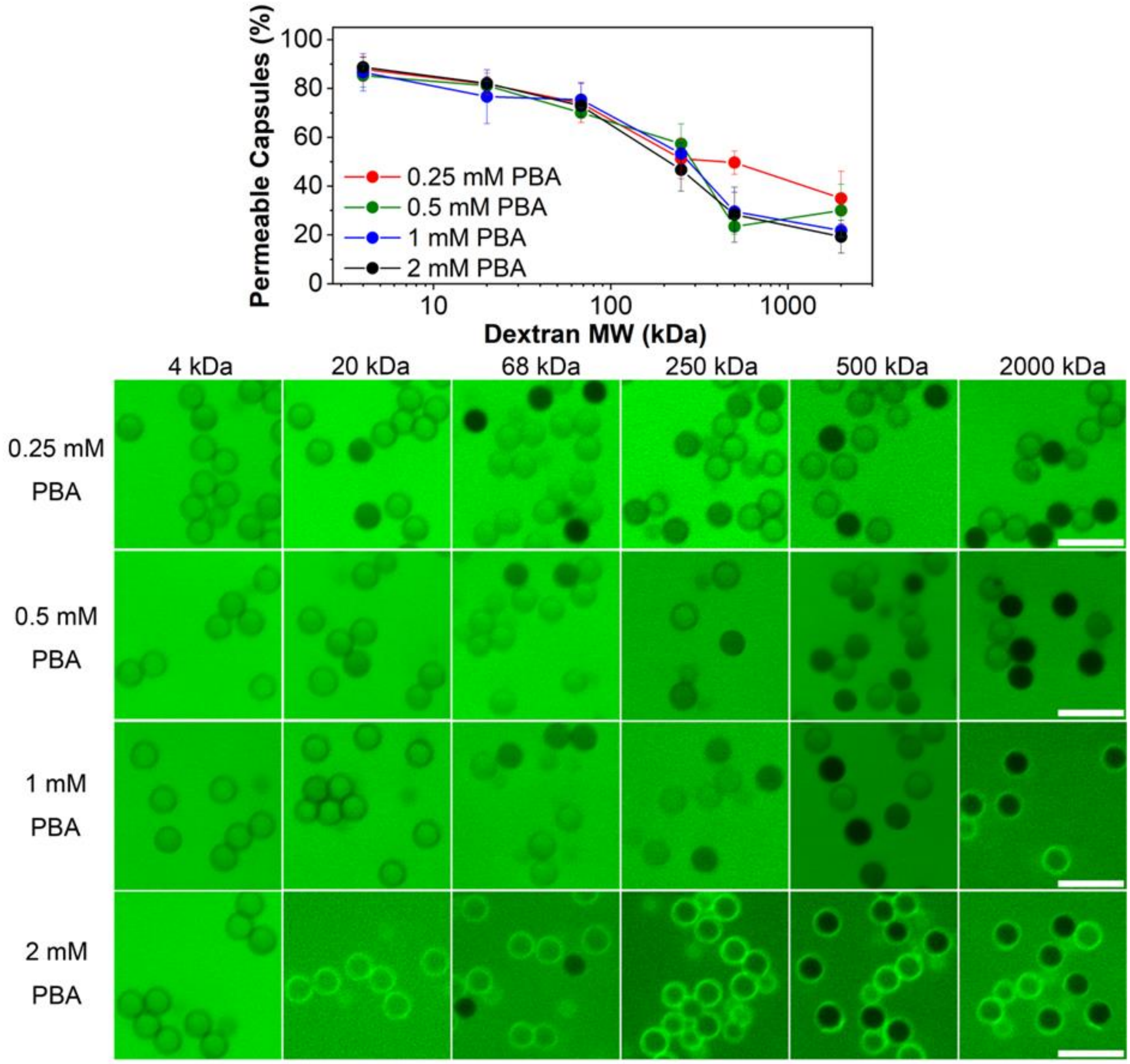

Figure S17. Comparison of the permeability of PBA_{118}–Fe^{III} MPN capsules to FITC-dextran of M_w ranging from 4 to 2000 kDa (above) and corresponding representative confocal microscopy images (below; scale bars are 5 µm). The PBA_{118}–Fe^{III} MPN capsules were prepared using different PBA concentrations in the assembly solution and a constant catechol/Fe^{III} ion ratio of 1:1. The permeability data are shown as the mean ± standard deviation of three independent experiments; 50–100 capsules were examined.

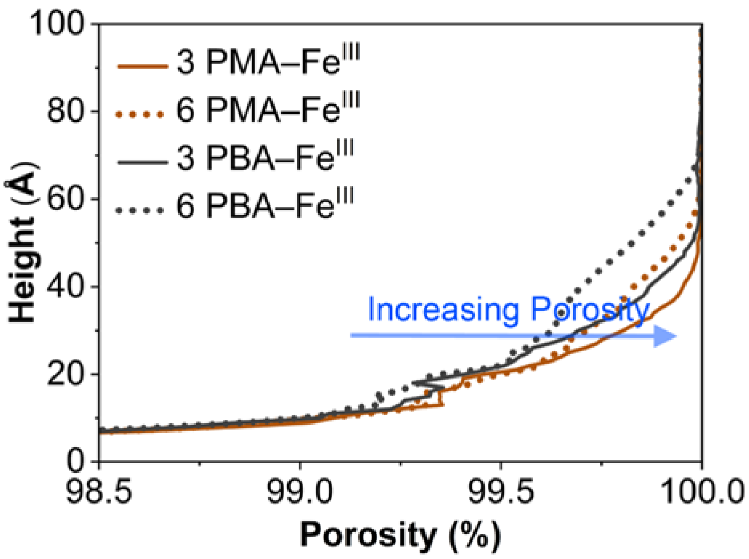

Figure S18. Porosity of the polymer–Fe^{III} films composed of 3 or 6 biscatechol-PMA and polymer–Fe^{III} films composed of 3 or 6 biscatechol-PBA as a function of height from the PS substrate.

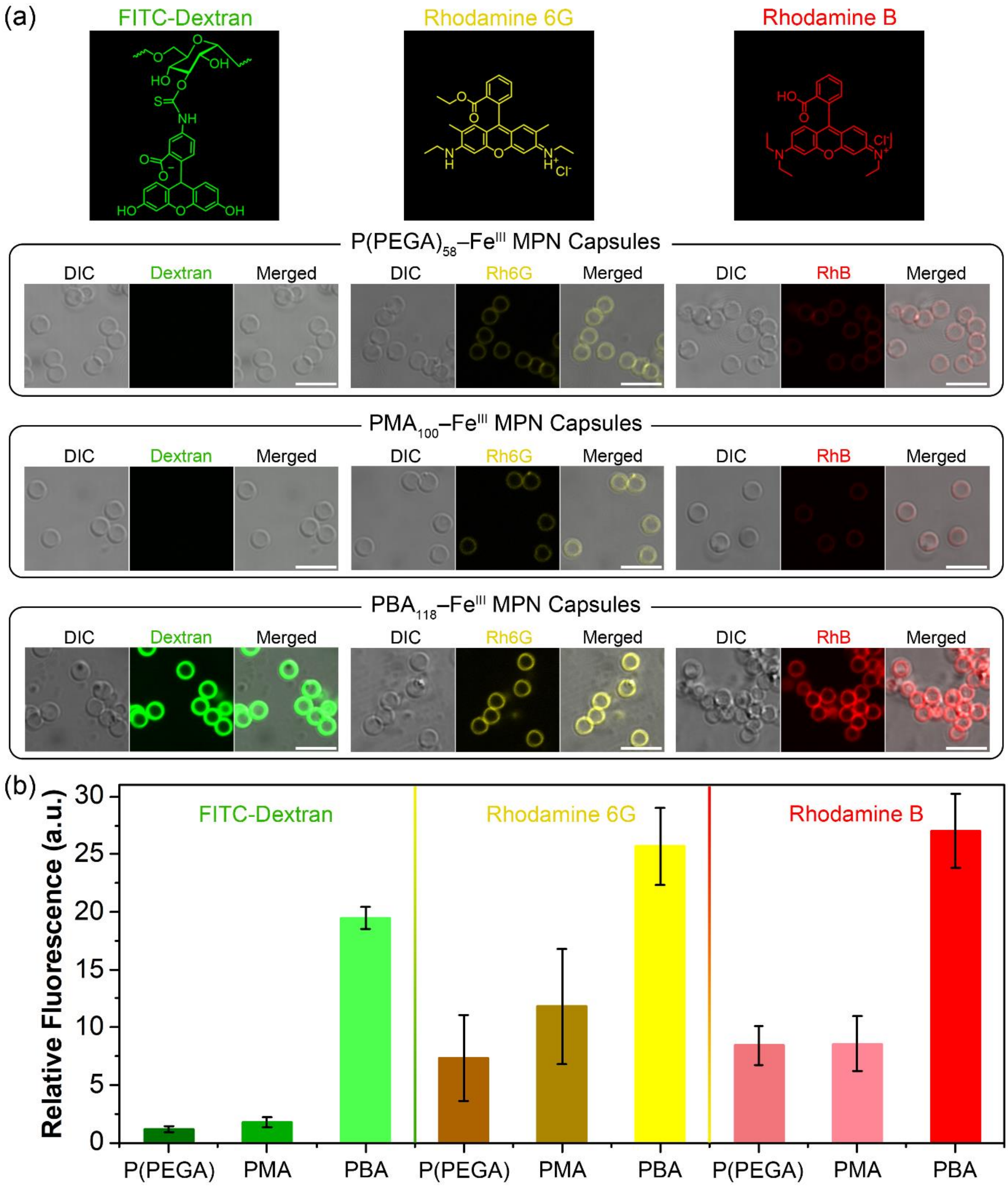

Figure S19. (a) DIC and CLSM images of 1.0 mM P(PEGA)$_{58}$–Fe^{III}, PMA$_{100}$–Fe^{III}, and PBA$_{118}$–Fe^{III} MPN capsules loaded with fluorescent FITC-dextran (green), rhodamine 6G (Rh6G, yellow), or rhodamine B (RhB, red); the fluorescent molecules were incubated for 5 min with the capsules. Scale bars are 5 μm. (b) Comparison of dye loading efficiencies into different MPN capsules.

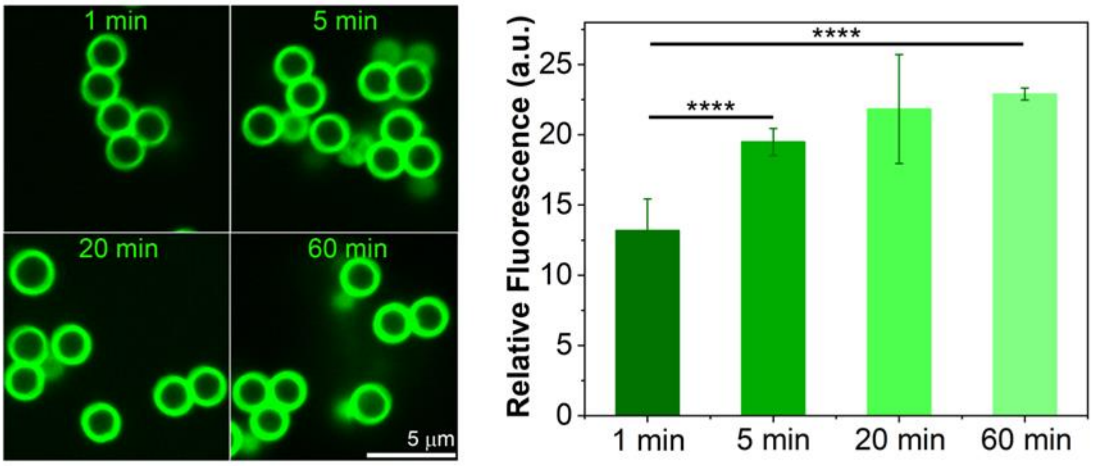

Figure S20. CLSM analysis of the loading of FITC-dextran into 1.0 mM PBA_{118}–Fe^{III} MPN capsules at varying incubation times (1–60 min). Statistical significance was determined by one-way ANOVA: **** $p < 0.0001$.

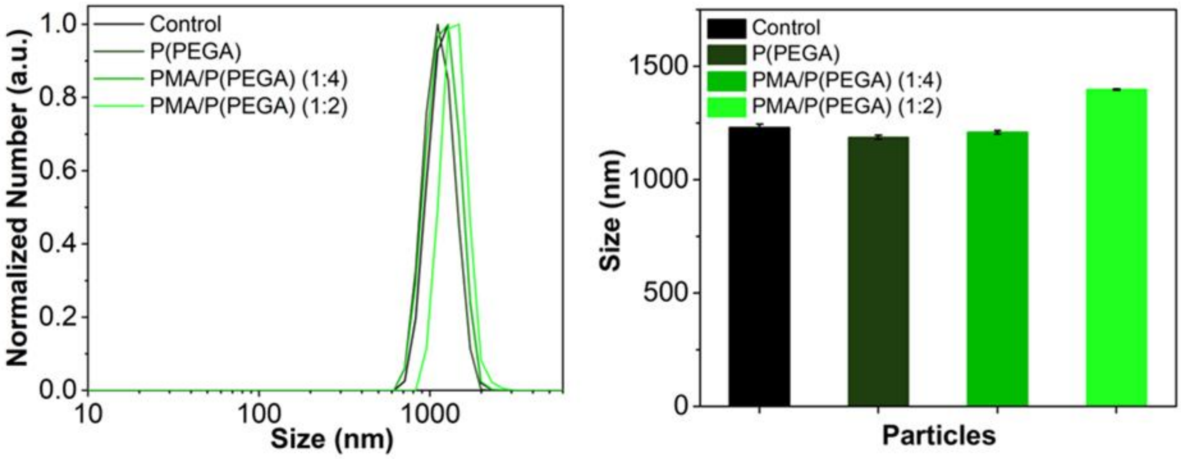

Figure S21. DLS analyses of green-fluorescent template particles (Control), and $P(PEGA)_{58}$–Fe^{III}, $PMA_{100}/P(PEGA)_{58}(1:4)$–$Fe^{III}$, and $PMA_{100}/P(PEGA)_{58}(1:2)$–$Fe^{III}$ MPN particles. The hydrodynamic diameters, determined by DLS (number size distribution), are shown as the average ± standard deviation of three measurements.

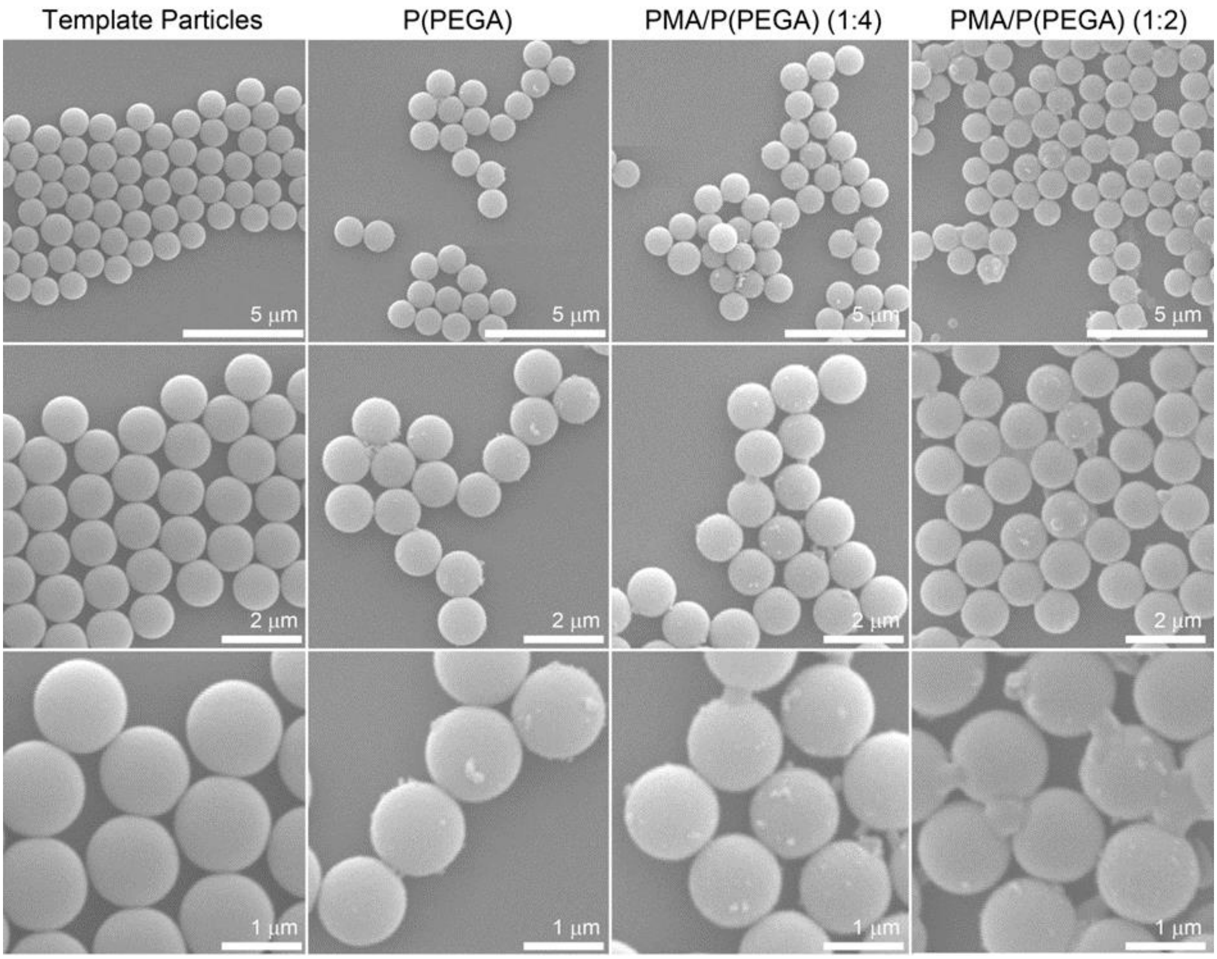

Figure S22. SEM images of green-fluorescent template particles, and $P(PEGA)_{58}$–Fe^{III}, $PMA_{100}/P(PEGA)_{58}$(1:4)–Fe^{III}, and $PMA_{100}/P(PEGA)_{58}$(1:2)–Fe^{III} MPN particles.

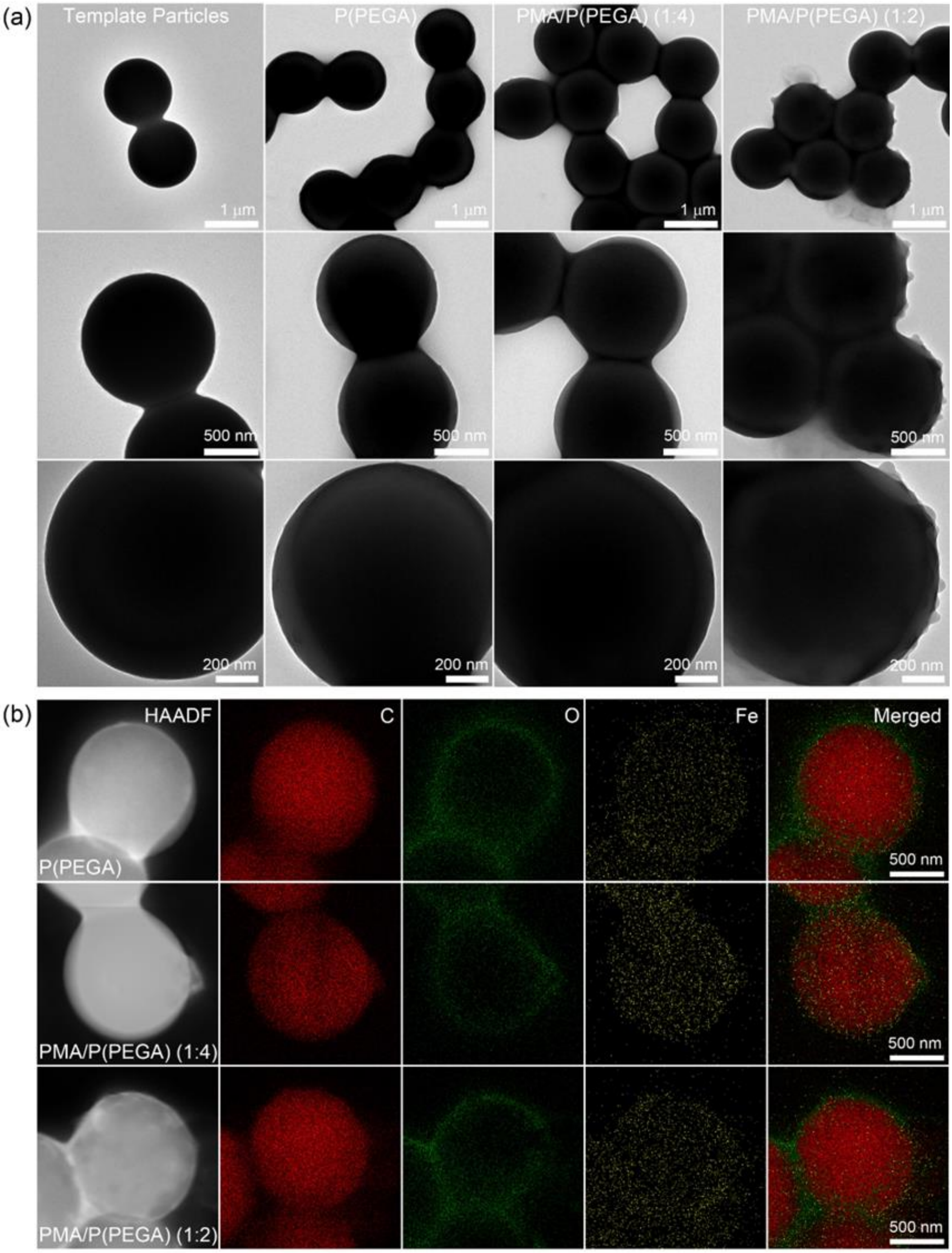

Figure S23. (a) TEM images of green-fluorescent template particles, and P(PEGA)$_{58}$–Fe^{III}, PMA$_{100}$/P(PEGA)$_{58}$(1:4)–Fe^{III}, and PMA$_{100}$/P(PEGA)$_{58}$(1:2)–Fe^{III} MPN particles. (b) HAADF and EDX elemental mapping of MPN particles.

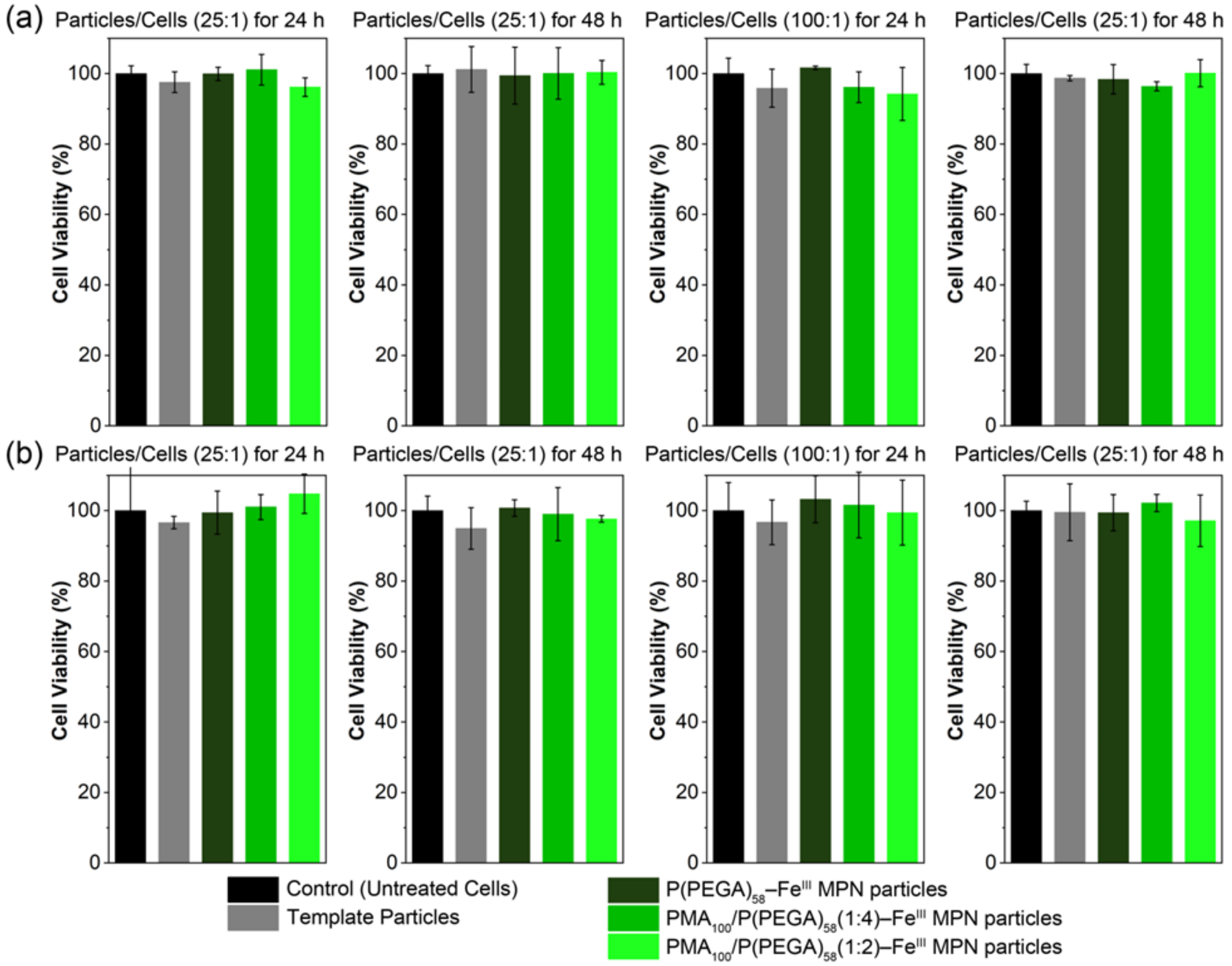

Figure S24. Cytotoxicity of template particles, and $P(PEGA)_{58}$–Fe^{III}, $PMA_{100}/P(PEGA)_{58}(1:4)$–$Fe^{III}$, and $PMA_{100}/P(PEGA)_{58}(1:2)$–$Fe^{III}$ MPN particles to (a) HeLa and (b) RAW 264.7 cells following incubation for 24 or 48 h in DMEM with 10% FBS at 37 °C; the particle-to-cell ratio was 25:1 or 100:1. For the cytotoxicity studies, nonfluorescent PS-COOH particles (1.86 μm) were used in the preparation of the MPN particles owing to the absorbance overlap at 475 nm that is observed between green-fluorescent particles used for the cell association experiments and the XTT method.

REFERENCES

[1] J. T. Lai, D. Filla, R. Shea, *Macromolecules* **2002**, *35*, 6754–6756.
[2] *Scienomics MAPS 4.3 Platform*, Scienomics SAS, Paris, 2020. https://www.scienomics.com/.
[3] W. C. Swope, H. C. Andersen, P. H. Berens, K. R. Wilson, *J. Chem. Phys.* **1982**, *76*, 637–649.
[4] S. Plimpton, *J. Comput. Phys.* **1995**, *117*, 1–19.
[5] Z. Lin, J. Zhou, C. Cortez-Jugo, Y. Han, Y. Ma, S. Pan, E. Hanssen, J. J. Richardson, F. Caruso, *J. Am. Chem. Soc.* **2020**, *142*, 335–341.
[6] a) N. C. Veitch, *Phytochemistry* **2004**, *65*, 249–259; b) M. Zhou, Z. Diwu, N. Panchuk-Voloshina, R. P. Haugland, *Anal. Biochem.* **1997**, *253*, 162–168.
[7] M. Faria, M. Bjornmalm, K. J. Thurecht, S. J. Kent, R. G. Parton, M. Kavallaris, A. P. R. Johnston, J. J. Gooding, S. R. Corrie, B. J. Boyd, P. Thordarson, A. K. Whittaker, M. M. Stevens, C. A. Prestidge, C. J. H. Porter, W. J. Parak, T. P. Davis, E. J. Crampin, F. Caruso, *Nat. Nanotechnol.* **2018**, *13*, 777–785.

Checklist
Minimum Information Reporting in Bio–Nano Experimental Literature

The MIRIBEL guidelines were introduced here: https://doi.org/10.1038/s41565-018-0246-4

The development of these guidelines was led by the ARC Centre of Excellence in Convergent Bio-Nano Science and Technology: https://www.cbns.org.au/. Any updates or revisions to this document will be made available here: http://doi.org/10.17605/OSF.IO/SMVTF. This document is made available under a CC-BY 4.0 license: https://creativecommons.org/licenses/by/4.0/.

The MIRIBEL guidelines were developed to facilitate reporting and dissemination of research in bio–nano science. Their development was inspired by various similar efforts:

- MIAME (microarray experiments): *Nat. Genet.* **29** (2001), 365; http://doi.org/10.1038/ng1201-365
- MIRIAM (biochemical models): *Nat. Biotechnol.* **23** (2005) 1509; http://doi.org/10.1038/nbt1156
- MIBBI (biology/biomedicine): *Nat. Biotechnol.* **26** (2008) 889; http://doi.org/10.1038/nbt.1411
- MIGS (genome sequencing): *Nat. Biotechnol.* **26** (2008) 541; http://doi.org/10.1038/nbt1360
- MIQE (quantitative PCR): *Clin. Chem.* **55** (2009) 611; http://doi.org/10.1373/clinchem.2008.112797
- ARRIVE (animal research): *PLOS Biol.* **8** (2010) e1000412; http://doi.org/10.1371/journal.pbio.1000412
- *Nature*'s reporting standards:
  - Life science: https://www.nature.com/authors/policies/reporting.pdf; e.g., *Nat. Nanotechnol.* **9** (2014) 949; http://doi.org/10.1038/nnano.2014.287
  - Solar cells: https://www.nature.com/authors/policies/solarchecklist.pdf; e.g., *Nat. Photonics* **9** (2015) 703; http://doi.org/10.1038/nphoton.2015.233
  - Lasers: https://www.nature.com/authors/policies/laserchecklist.pdf; e.g., *Nat. Photonics* **11** (2017) 139; http://doi.org/10.1038/nphoton.2017.28
- The "TOP guidelines": e.g., *Science* **352** (2016) 1147; http://doi.org/10.1126/science.aag2359

Similar to many of the efforts listed above, the parameters included in this checklist are **not** intended to be definitive requirements; instead they are intended as 'points to be considered', with authors themselves deciding which parameters are—and which are not—appropriate for their specific study.

This document is intended to be a living document, which we propose is revisited and amended annually by interested members of the community, who are encouraged to contact the authors of this document. Parts of this document were developed at the annual International Nanomedicine Conference in Sydney, Australia: http://www.oznanomed.org/, which will continue to act as a venue for their review and development, and interested members of the community are encouraged to attend.

After filling out the following pages, this checklist document can be attached as a "Supporting Information" document during submission of a manuscript to inform Editors and Reviewers (and eventually readers) that all points of MIRIBEL have been considered.

Supplementary Table 1. Material characterization*

Question	Yes	No
1.1 Are "**best reporting practices**" **available** for the nanomaterial used? For examples, see *Chem. Mater.* **28** (2016) 3535; http://doi.org/10.1021/acs.chemmater.6b01854 and *Chem. Mater.* **29** (2017) 1; http://doi.org/10.1021/acs.chemmater.6b05235		√
1.2 If they are available, **are they used**? If not available, ignore this question and proceed to the next one.		
1.3 Are extensive and clear instructions reported detailing all steps of **synthesis** and the resulting **composition** of the nanomaterial? For examples, see *Chem. Mater.* **26** (2014) 1765; http://doi.org/10.1021/cm500632c, and *Chem. Mater.* **26** (2014) 2211; http://doi.org/10.1021/cm5010449. Extensive use of photos, images, and videos are strongly encouraged. For example, see *Chem. Mater.* **28** (2016) 8441; http://doi.org/10.1021/acs.chemmater.6b04639	√	
1.4 Is the **size** (or **dimensions**, if non-spherical) and **shape of** the nanomaterial reported?	√	
1.5 Is the **size dispersity** or **aggregation** of the nanomaterial reported?	√	
1.6 Is the **zeta potential** of the nanomaterial reported?	√	
1.7 Is the **density (mass/volume)** of the nanomaterial reported?		√
1.8 Is the amount of any **drug loaded** reported? 'Drug' here broadly refers to functional cargos (e.g., proteins, small molecules, nucleic acids).	not applicable	
1.9 Is the **targeting performance** of the nanomaterial reported, including **amount** of ligand bound to the nanomaterial if the material has been functionalised through addition of targeting ligands?	not applicable	
1.10 Is the **label signal** per nanomaterial/particle reported? For example, fluorescence signal per particle for fluorescently labelled nanomaterials.		√
1.11 If a material property not listed here is varied, has it been **quantified**?		√
1.12 Were characterizations performed in a **fluid mimicking biological conditions**?		√
1.13 Are details of how these parameters were **measured/estimated** provided?	√	
Explanation for **No** (if needed):<br>1.12: Particles were characterized by DIC microscopy in aqueous solution.		

***Ideally, material characterization should be performed in the same biological environment as that in which the study will be conducted. For example, for cell culture studies with nanoparticles, characterization steps would ideally be performed on nanoparticles dispersed in cell culture media. If this is not possible, then characteristics of the dispersant used (e.g., pH, ionic strength) should mimic as much as possible the biological environment being studied.

Supplementary Table 2. Biological characterization*

**Question**	**Yes**	**No**
2.1 Are **cell seeding details**, including **number of cells plated, confluency at start of experiment,** and **time between seeding and experiment** reported?	√	
2.2 If a standardised cell line is used, are the **designation and source** provided?	√	
2.3 Is the **passage number** (total number of times a cell culture has been subcultured) known and reported?	√	
2.4 Is the last instance of **verification of cell line** reported? If no verification has been performed, is the time passed and passage number since acquisition from trusted source (e.g., ATCC or ECACC) reported? For information, see *Science* **347** (2015) 938; http://doi.org/10.1126/science.347.6225.938		√
2.5 Are the results from **mycoplasma testing** of cell cultures reported?	√	
2.6 Is the **background signal of cells/tissue** reported? (E.g., the fluorescence signal of cells without particles in the case of a flow cytometry experiment.)	√	
2.7 Are **toxicity studies** provided to demonstrate that the material has the expected toxicity, and that the experimental protocol followed does not?	√	
2.8 Are details of media preparation (**type of media, serum,** any **added antibiotics**) provided?	√	
2.9 Is a **justification of the biological model** used provided? For examples for cancer models, see *Cancer Res.* **75** (2015) 4016; http://doi.org/10.1158/0008-5472.CAN-15-1558, and *Mol. Ther*. **20** (2012) 882; http://doi.org/10.1038/mt.2012.73, and *ACS Nano* **11** (2017) 9594; http://doi.org/10.1021/acsnano.7b04855	not applicable	
2.10 Is characterization of the **biological fluid** (*ex vivo*/*in vitro*) reported? For example, when investigating protein adsorption onto nanoparticles dispersed in blood serum, pertinent aspects of the blood serum should be characterised (e.g., protein concentrations and differences between donors used in study).	not applicable	
2.11 For **animal experiments**, are the ARRIVE guidelines followed? For details, see *PLOS Biol.* **8** (2010) e1000412; http://doi.org/10.1371/journal.pbio.1000412	not applicable	
Explanation for **No** (if needed):<br>2.4: Cells were purchased from the ATCC. The passage number was reported and regular mycoplasma test was conducted.		

*For *in vitro* experiments (e.g., cell culture), *ex vivo* experiments (e.g., in blood samples), and *in vivo* experiments (e.g., animal models). The questions above that are appropriate depend on the type of experiment conducted.

Supplementary Table 3. Experimental details*

**Question**	**Yes**	**No**
3.1 For cell culture experiments: are **cell culture dimensions** including **type of well**, **volume of added media**, reported? Are cell types (i.e.; adherent vs suspension) and **orientation** (if non-standard) reported?	√	
3.2 Is the **dose of material administered** reported? This is typically provided in nanomaterial mass, volume, number, or surface area added. Is sufficient information reported so that regardless of which one is provided, the other dosage metrics can be calculated (i.e. using the dimensions and density of the nanomaterial)?	√	
3.3 For each type of imaging performed, are details of how **imaging** was performed provided, including details of **shielding, non-uniform image processing**, and any **contrast agents** added?	√	
3.4 Are details of how the dose was administered provided, including **method of administration, injection location, rate of administration,** and details of **multiple injections**?	not applicable	
3.5 Is the methodology used to **equalise dosage** provided?	√	
3.6 Is the **delivered dose** to tissues and/or organs (in vivo) reported, as % injected dose per gram of tissue (%ID g^{-1})?	not applicable	
3.7 Is **mass of each organ/tissue measured** and **mass of material** reported?	not applicable	
3.8 Are the **signals of cells/tissues with nanomaterials** reported? For instance, for fluorescently labelled nanoparticles, the total number of particles per cell or the fluorescence intensity of particles + cells, at each assessed timepoint.	√	
3.9 Are **data analysis details**, including **code used** for analysis provided?	√	
3.10 Is the **raw data** or **distribution of values** underlying the reported results provided? For examples, see *R. Soc. Open Sci.* **3** (2016) 150547; http://doi.org/10.1098/rsos.150547, https://opennessinitiative.org/making-your-data-public/, http://journals.plos.org/plosone/s/data-availability, and https://www.nature.com/sdata/policies/repositories	not applicable	
Explanation for **No** (if needed):		

* The use of protocol repositories (e.g., *Protocol Exchange* http://www.nature.com/protocolexchange/) and published standard methods and protocols (e.g., *Chem. Mater*. **29** (2017) 1; http://doi.org/10.1021/acs.chemmater.6b05235, and *Chem. Mater*. **29** (2017) 475; http://doi.org/10.1021/acs.chemmater.6b05481) are encouraged.
